# Acquired resistance to EGFR-TKIs in NSCLC mediates epigenetic downregulation of MUC17 by facilitating NF-κB activity via UHRF1/DNMT1 complex

**DOI:** 10.7150/ijbs.75963

**Published:** 2023-01-09

**Authors:** Shuye Lin, Hongyun Ruan, Lin Qin, Cong Zhao, Meng Gu, Ziyu Wang, Bin Liu, Haichao Wang, Jinghui Wang

**Affiliations:** 1 Cancer Research Center, Beijing Chest Hospital, Capital Medical University, Beijing Tuberculosis and Thoracic Tumor Research Institute, Beijing 101149, China.; 2 Institute of Resources and Environment, Beijing Academy of Science and Technology, Beijing, 100089, China.; 3 Department of Endoscopic Diagnosis and Treatment, Beijing Chest Hospital, Capital Medical University, Beijing Tuberculosis and Thoracic Tumor Institute, Beijing 101149, China.

**Keywords:** DNA methylation, mucin 17, myeloid zinc-finger 1, NF-κB, EGFR-TKIs resistance

## Abstract

Treatment with epidermal growth factor receptor (EGFR) tyrosine kinase inhibitors (TKIs) has brought significant benefits to non-small cell lung cancer (NSCLC) patients with EGFR mutations. However, most patients eventually develop acquired resistance after treatment. This study investigated the epigenetic effects of mucin 17 (MUC17) in acquired drug-resistant cells of EGFR-TKIs. We found that GR/OR (gefitinib/osimertinib-resistance) cells enhance genome-wide DNA hypermethylation, mainly in 5-UTR associated with multiple oncogenic pathways, in which GR/OR cells exerted a pro-oncogenic effect by downregulating mucin 17 (MUC17) expression in a dose- and time-dependent manner. Gefitinib/osimertinib acquired resistance mediated down-regulation of MUC17 by promoting DNMT1/UHRF1 complex-dependent promoter methylation, thereby activating NF-κB activity. MUC17 increased the generation of IκB-α and inhibit NF-κB activity by promoting the expression of MZF1. *In vivo* results also showed that DNMT1 inhibitor (5-Aza) in combination with gefitinib/osimertinib restored sensitivity to OR/GR cells. Acquired drug resistance of gefitinib/osimertinib promoted UHRF1/DNMT1 complex to inhibit the expression of MUC17. MUC17 in GR/OR cells may act as an epigenetic sensor for biomonitoring the resistance to EGFR-TKIs.

## Introduction

Lung cancer is the second most commonly diagnosed cancer and remains the leading cause of cancer death worldwide, with an estimated 2.2 million new cases and 1.8 million deaths in 2020 1. Non-small cell lung cancer (NSCLC) is the most common histological type of lung cancer, accounting for about 85% of lung cancer patients 2. EGFR mutations, as significant oncogenic drivers in NSCLC, open the curtain on a biomarker-oriented treatment model for patients with advanced disease 3. Many EGFR tyrosine kinase inhibitors (TKIs) have been developed, including the commonly used first-generation TKIs gefitinib and the highly effective third-generation TKIs osimertinib. Despite their remarkable efficacy in the treatment of patients, resistance to these drugs remains a fundamental challenge that has yet to be addressed. At present, there are two mainstream viewpoints on acquired resistance of EGFR-TKIs, namely, changes of targeted kinases (such as EGFR C797S mutation 4-6) and changes in downstream signal pathways of the design target (such as BRAF fusions, KRAS mutations, NRAS mutations, and MAP2K1 mutations in the RAS-MAPK pathway 7-10). However, most of these studies have focused on genetic changes in genes associated with EGFR. Epigenetic changes frequently occur in patients with poor treatment responses to TKIs, and modulating them enhance the cytotoxic effects of antitumor therapy 11, 12. Compared with permanent gene mutation, epigenetic changes controlling gene expression plays an important role in regulating cell differentiation and malignant transformation in a gradual and modifiable manner after cell resistance, so it may use as a potential sensitive marker for biological monitoring of drug resistance 13, 14.

Mucins (MUCs) are a group of large glycoproteins expressed by various epithelial cells, which form the protective component of the airway mucosal barrier and are the first barrier for human contact with external substances. Some MUCs preferentially distribute in the respiratory tract and lung not only to control the local molecular environment but also to contribute to cellular signal transduction in response to external stimuli 15-17. However, members of the mucin family play different roles in drug resistance and tumor progression. On the one hand, the high expression of MUCs induces PD-L1 to promote immune escape and makes cells develop drug resistance 18, 19. On the other hand, inflammatory factors induce expression of MUCs. High expression of MUCs inhibits the activation of NF-κB, which inhibits tumor progression in response to pro-inflammatory cytokines 20. MUCs have been reported to be closely related to EGFR-TKIs resistance 18, 19, 21. The dual role of MUCs in drug resistance and tumor progression may depend on the different responses of different members of the mucin family to various drug-resistant microenvironments, indicating that this complex relationship remains to be determined.

Our previous studies showed that reduced expression of membrane-bound mucins (MUC17 and MUC22) modulated decreasing expression of NF-κB inhibitor α (IκB-α) in cancer cells and were associated with poor prognosis 22, 23. Recent studies have demonstrated constitutive activation of NF-κB in the acquired resistance to EGFR-TKIs 24, 25. The hyperactivation of NF-κB promotes the occurrence of tumor by promoting the proliferation of cancer cells and producing drug resistance 24, 26. Inhibition of NF-κB improves sensitivity to EGFR-TKIs and decreases resistance-induced oncogenic functions 25, 26. However, MUCs are membrane protein that is unlikely to regulate IκB-α activity by direct binding to its promoter. Therefore, the precise link between MUCs and NF-κB remains to be elucidated. MZF1 belongs to Krüppel-like family of transcription factors and is involved in cell proliferation and differentiation 27-29. MZF1 has been shown to be essential for the regulation of proliferation, migration and invasion of malignant tumor cells 30-35. As a transcription factor, MZF1 is well-known to regulate the expression of IκB-α 36. Therefore, we investigated the regulatory mechanism of MZF1 on EGFR-TKIs resistant cells and its relationship with MUCs-NF-κB signal.

The genomic instability and epigenetic changes of EGF-TKIs resistant cells are related to tumor malignancy and cell resistance. Therefore, we studied the effect of EGFR-TKIs resistance on the growth and metastasis of lung cancer cells associated with DNA methylation. We found an effect of EGFR-TKIs resistance on DNA methylation in favor of lung carcinogenesis via multiple tumor progression pathways, among which the specific downregulation of MUC17 by UHRF1/DNMT1-mediated promoter methylation promote the activity of NF-κB by inhibiting the expression of MZF1, has implications in evaluating drug resistance.

## Materials and methods

### Oligonucleotides, Antibodies and Reagents

All oligonucleotide sequences and cDNA clones used to construct recombinant plasmids and the antibodies and reagents used in this study are listed in **Supplementary Table 1, Supplementary Table 2** and **Supplementary Table 3**.

### Cell culture

The human NSCLC cell line HCC827, PC9, A549 and NCI-H1299 were obtained from ATCC. All cell lines were authenticated by the short tandem repeat (STR) method. *TransSafe^TM^* Mycoplasma Prevention Reagent (TransGene, China) was used to prevent mycoplasma contamination. HCC827 and PC9 were exposed to increasing concentrations of gefitinib (5nM to 1μΜ) or osimetinib (1nM to 500nM) as previously described^37^. A549 and NCI-H1299 were exposed to different concentrations of gefitinib (5nM to 4μΜ) or osimetinib (1nM to 4μM) and established resistant cell lines (**Supplementary Figure 1**). Cell lines were divided into low-density (30% confluence) and grown in 90% RPMI 1640 (Gibco) containing 10% fetal bovine serum (Gibco).

### Patients and Specimens

This study was approved by the Ethics Committee of the Beijing Chest Hospital (JYS-2021-013). All patients provided their informed consent. The cohort consisted of 57 patients, and samples included 10 from patients with chronic pneumonia (CP), 22 from patients before treatment (BT), 13 with gefitinib-resistance (GR) and 12 with osimertinib-resistance (OR) (**Table 1**). BALF specimens and in situ tissue samples were obtained through bronchoscopy or radical resection, respectively. BALF specimens were collected using the QIAsymphony PAXgene Blood ccfDNA Kit (QIAGEN, USA). The tissue samples were snap-frozen in liquid nitrogen in preparation for RNA extraction. Clinicopathological data was obtained from clinical and histopathological reports. Histological evaluation of the specimens was carried out by two senior pathologists. The severity of NSCLC was classified according to the 8^th^ edition tumor staging system recommended by the American Joint Committee on Cancer. Information on the tissue and BALF specimens used for the analysis of *MUC17* mRNA expression and methylation is listed in **Supplementary Table 4**.

### RNA isolation and quantitative polymerase chain reaction (qPCR)

The well-stated cells were selected, and TRIzol reagent (Invitrogen, Grand Island, NY, USA) was added at 10^6^ cells/mL. First strand cDNA was synthesized with the Superscript First-Strand Synthesis System (Invitrogen). qPCR was performed using 2× SYBR Green-based qPCR reagents (Applied Biosystems, California, USA) and analyzed by an ABI 7500 fast qPCR machine. The standard curve was used to analyze the data, and the dissolution curve ensured the specificity of the products. The primers used are listed in **Supplementary Table 1**. The relative expression of each gene was standardized using the 2^-ΔΔCt^ method.

### DNA extraction, bisulfite modification, methylation-specific qPCR (MSP-qPCR) and MassARRAY

Genomic DNA was extracted by the QIAamp DNA Mini Kit (QIAGEN, Valencia CA, USA). Bisulfite modification of DNA was performed using a Zymo DNA Methylation Kit (Zymo Research, Irvine, CA, USA), and then the DNA was detected by qPCR. The methylation of *MUC17* CpG sites was also detected by MassArray (Oebiotech, Beijing). The primers used are listed in **Supplementary Table 1**. The relative expression of each gene was standardized using the 2^-ΔΔCt^ method.

### Whole genome bisulfite sequencing (WGBS) and global DNA methylation assay

The extracted complete genomic DNA was shipped to BGI (Beijing Genomics Institute) in Wuhan, China. Bisulfite-medicated DNA was analyzed using Illumina's Infinium according to the manufacturer's protocol. Whole-genome bisulfite sequencing was used to detect the change in single-based resolution and the relative number of 5-mCs across the genome of A549, A549/GR and A549/OR cells (**Supplementary Table 5**). R (http://www.r-project.org) was used to process and analyze the raw data.

Genomic DNA was extracted by the QIAamp DNA Mini Kit (QIAGEN, Valencia CA, USA). The quantification of global DNA methylation was performed using a Global DNA Methylation Assay Kit (5 Methyl Cytosine, Colorimetric, ab233486) for the quantification of global DNA methylation. The results were detected by a dual wavelength of 450 nm.

### Western blot analysis

The well-grown cells were inoculated into a 60 mm^2^ culture dish. When cell fusion reached 80%, the cells were lysed with RIPA lysis buffer. Cell membrane, cytoplasm and nucleus were separated using Cotoplasimc-Nuclear protein separation kit (Aoqing Biotechnology, Beijing, China). After quantifying the samples, 30 μg/well was used to calculate the amount of sample added. Proteins were resolved by sodium dodecyl sulfate-polyacrylamide gel electrophoresis (SDS-PAGE) and transferred onto PVDF membranes using a Bio-Rad Mini PROTEAN 3 system (Hercule, CA). The membranes were blocked with PBS containing 5% milk and 0.1% Tween-20 at room temperature for 1 hour. The samples were incubated with the primary antibody at 4 °C overnight. The anti-mouse or anti-rabbit horseradish peroxidase-labeled secondary antibody was added and incubated for 1h (**Supplementary Table 2**). An ECL chemiluminescence kit was added for color development. A smart gel image analysis system was used for development.

### Enzyme-linked immunosorbent assay (ELISA)

Cells were added to a 6-well cell culture plate at 1×10^6^ cells/well. The cells were lysed with RIPA lysis buffer. The antibody was diluted to a protein content of 1-10 μg/mL with carbonate buffer (pH 9.6) in a 96-well ELISA plate. Then the samples were incubated at 37 °C with 5% milk and incubated MUC17 antibody or positive serum and negative serum was added and incubated at 37 °C for 1 h. The anti-mouse or anti-rabbit horseradish peroxidase-labeled secondary antibody was added and incubated for 1 h and detected with 100 μL of TMB (e4849, TIANGEN, China) for 15 min at 37 °C away from light. The reaction was then stopped by the addition of 50 μL of 3 M H_2_SO4. The results were detected by a dual wavelength of 450 nm. The optimal condition was obtained by comparing the positive/negative ratio (P/N > 2.1 is positive) of the samples.

### Immunofluorescence staining (IF)

Cells were dropped on cover glass, cultured in 6-well cell culture plates for 24 h, and fixed with 4% paraformaldehyde. The membranes were blocked with PBS containing 5% BSA. Primary antibodies were incubated overnight at 4 °C (**Supplementary Table 2**). Secondary antibodies were added and incubated at room temperature for 1 h. DAPI (10 μg/ml in PBS, Invitrogen, USA) staining was performed and the cell localization of protein molecules was observed with Laser confocal microscopy (Leica Sp5 Laser Scanning Confocal Microscope, GE).

### Immunohistochemistry (IHC)

IHC was performed on 5 mm thick serial sections of formaldehyde fixed. The sections were incubated with antibodies overnight at 4°C (**Supplementary Table 2**). The sections were incubated with DAB, and then were analyzed by Vectra3 automated quantitative pathology system from PerkinElmer Inc. (Boston, MA, USA). Patient tumor specimens were analyzed and scores described previously 38, 39.

### Luciferase assay

Luciferase reporter assay was performed using a Dual-Luciferase Reporter Assay System (Promega, Madison, WI, USA) with the pGL3 basic luciferase reporter system. The luciferase reporter containing *MUC17* and *IκB-α* promoter region was constructed and transfected into HCC827, HCC827/GR and HCC827/OR cells as previously described 20, 38. *MUC17* promoter-specific luciferase fragments were inserted into the *KpnI/XhoI* sites and *IκB-α* promoter-specific luciferase fragments were construct into *BglII/HindIII* sites of the pGL3-Basic reporter, with the pRL-TK vector (RRID: Addgene_11313) used as an internal control. The primers used for construction of the plasmid are listed in **Supplementary Table 1.** Promoter-specific luciferase constructs and controls were transfected with into cells using Lipofectamine 2000 (Invitrogen). The luciferase signal was first normalized to the control luciferase signal and then normalized to the signal from control group.

### Chromatin immunoprecipitation (ChIP) and methylated DNA immunoprecipitation (MeDIP)

HCC827 and A549 cells were fixed with 1% formaldehyde. Chromatin was prepared by sonication of cell lysate and preclearing with protein A beads. Aliquots of precleared chromatin solution, named IP fractions, were incubated with 2 μg of specific antibody or preimmune rabbit IgG on a rotation platform at 4 °C overnight. One percent of the IP fraction served as the ChIP input control. The antibody-enriched protein-DNA complexes were precipitated with protein A beads from the IP fractions. DNA fragments were released by reverse crosslinking and purified by using a QIAquick purification kit (QIAGEN, Valencia CA, USA). Immunoprecipitated DNA fractions were analyzed by qPCR.

Genomic DNA was extracted from HCC827 and A549 cells and prepared by sonication. The sonicated DNA was then immunoprecipitated with a monoclonal antibody against 5-methylcytidine (5mC) at 4 °C overnight. A portion of the sonicated DNA was left untreated to serve as an input control. DNA fragments were released by reverse crosslinking and purified by using a QIAquick purification kit (QIAGEN, Valencia CA, USA). Immunoprecipitated DNA fractions were analyzed by qPCR.

### Cell viability and colony formation

Cells were added to a 96-well cell culture plate at 2×10^3^ cells/well and 6 wells per well. The cells were observed continuously for 4 days. MTT (Invitrogen, USA) was added to the cell culture medium daily to achieve a final concentration of 5 μg/mL. The results were detected by a dual wavelength of 490/570 nm.

Cells were inoculated into a 6-well cell culture plate with 100 cells per well, and the medium was changed every three days. After the cell clones grew to the size visible to the naked eye, cells were fixed in 4% paraformaldehyde. 0.5% crystal violet was dyed and then photographed.

### Migration assay

Transwells with 8-μm pore membranes were obtained from Corning Inc. The upper chamber was inoculated with 400 µL serum-free medium containing 2×10^4^ cells. The lower cavity was filled with 500 µl RPMI 1640 with 10% FBS. After 24 hours, the translocated cells were fixed in 4% paraformaldehyde for 30 min and stained with 0.5% crystal violet (Beyotime).

### DNA constructs and transfection

Truncated* MUC17* (*MUC17*) was cloned into pcDNA 3.1 (Invitrogen), and short hairpin RNA against *MUC17* was cloned into pSilencer 3.0 (Invitrogen) 22. They were constructed by GeneChem Co. (Shanghai, China) and transfected into HCC827 and PC9 cells using Lipofectamine 2000 according to the manufacturer's instructions (Invitrogen).

### Tumorigenicity

The animal handling and all in vivo experimental procedures were approved by the Institutional Animal Ethics Committee of Beijing Chest Hospital. To observe the effect of EGFR-TKIs resistance on tumor growth in vivo, HCC827, HCC827/GR and HCC827/OR cells (2 × 10^6^) in 0.1 ml of PBS were subcutaneously injected in both flanks of 4-week-old Balb/c female athymic mice (Vital River Laboratories, Beijing, China). Tumor diameters and body weights in NSCLC xenograft tumor-bearing nude mice were measured and documented every 6 days until the animals were sacrificed at day 30.NCI-H1299, NCI-H1299/GR and NCI-H1299/OR cells (1×10^6^) in 0.1 ml PBS were subcutaneously implanted in 4-week-old BALB/c female athymic mice (Vital River Laboratories, Beijing, China) with both flank injections. After 1 week injection, mice were treated with EGFR-TKIs and/or 5-Aza **(Figure 7A)**. Tumor diameters and body weights in NSCLC xenograft tumor-bearing nude mice were measured and documented every 4 days until the animals were sacrificed at day 24. NSCLC tumor xenografts were isolated and weighed. Tumor volume was calculated by measuring the longest (a) and shortest (b) diameters of the tumor and calculated by the formula ab2/2, where a is the longest diameter and b is the shortest diameter.

### Statistical analysis

The data are expressed as the means ± standard deviation (SD) of at least three independent experiments. The qPCR, MTT and clone formation results were analyzed by Student's t test or one-way analysis of variance (ANOVA) with Tukey's post-hoc test. KEGG pathway and Gene Ontology enrichment analyses were performed using Fisher's exact test for significance. *p < 0.05 indicates a significant difference, **p < 0.01 indicates an extremely significant difference. The above statistical analysis was performed with SPSS 23.0 software.

## Results

### Acquired resistance to EGFR-TKIs promotes the tumorigenic function of NSCLC

**Figure 1:**
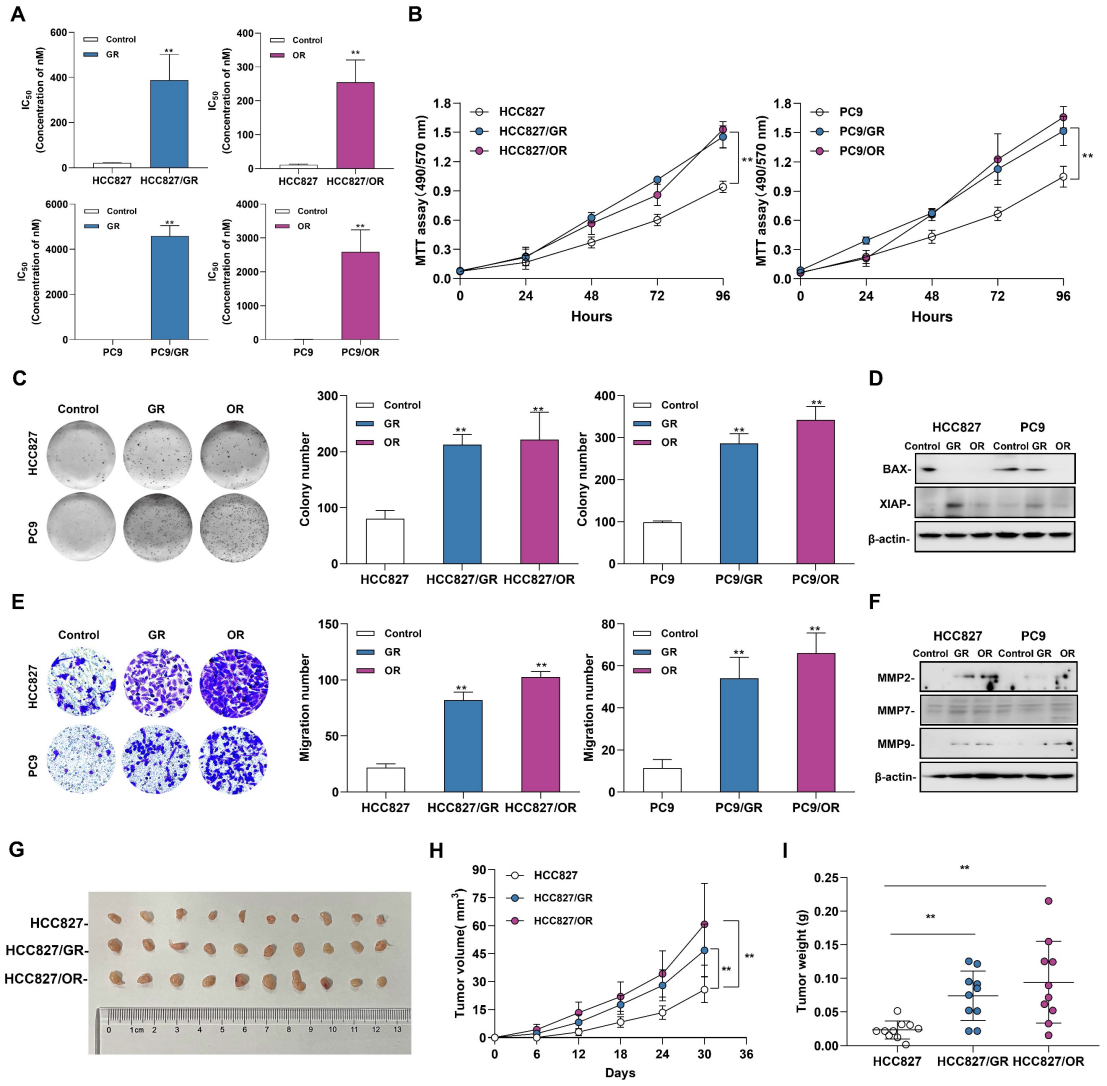
** The Biological function of gefitinib/osimertinib-resistant cells.** HCC827 and PC9 cells were treated with gefitinib/osimertinib to establish gefitinib-resistant cells (GR) and osimertinib-resistant cells (OR). **(A)** IC50 analysis of gefitinib /Osimertinib-resistant cells. **(B)** MTT assay performed to monitor the viability of cells over 4 days. **(C)** Quantification of the total number of colonies formed for 2 weeks. **(D)** Western blotting analysis of the protein levels of BAX and XIAP in GR and OR. β-actin serves as the loading control. **(E)** Quantification of cell motility by Transwell assay. **(F)** Western blotting analysis of the protein levels of MMP2, MMP7 and MMP9 in GR and OR. β-actin serves as the loading control. **(G)-(I)** The effect of gefitinib/osimertinib-resistants on lung tumorigenicity. HCC827, HCC827/GR and HCC827/OR cells were transplanted subcutaneously into the left and right flanks of nude mice (n = 2 flanks × 5 mice in each group). **(G)** Images of dissected HCC827 xenograft tumors from nude mice on day 30. **(H)** Tumor volumes of tumor-bearing mice measured at the indicated time points. **(I)** Tumor weights of the mouse xenografts. Data are presented as the mean ± standard deviation (n=3). One-way ANOVA with Tukey's post-hoc test was performed. *p < 0.05, ** *p* < 0.01, significant differences versus control.

To evaluate the effects of acquired resistance to gefitinib/osimertinib on the biological functions in tumor cells, we first measured the IC50 of gefitinib-resistant cells (GR) and osimertinib-resistant cells (OR), and the data showed a significantly increase in IC50 after resistant to EGFR-TKIs inhibitors **(Figure 1A and Supplementary Figure 2A)**. In addition, MTT assay confirmed that the proliferation rate of drug-resistant cells induced by gefitinib/osimertinib was significantly increased in both NSCLC cells **(Figure 1B and Supplementary Figure 2B)**. Colony formation assay further confirmed the proliferation-promoting effect of gefitinib/osimertinib on drug-resistant cells, showing that the OR/GR formed more colonies than the control cells **(Figure 1C and Supplementary Figure 2C)**. At the same time, Western blotting also detected a decrease in BAX and an increase in XIAP expression **(Figure 1D and Supplementary Figure 2D)**, suggesting that gefitinib/osimertinib-induced acquired resistance could promote cell proliferation. Transwell analysis further showed a significant increase in the number of migrating cells in OR/GR cells compared with the control **(Figure 1E and Supplementary Figure 2E)**. Western blotting demonstrated that the expression of matrix metallopeptidases 2, 7, and 9 (MMP2, MMP7, and MMP9), three important regulators of cancer invasion and metastasis, was markedly increased in GR and OR cells** (Figure 1F and Supplementary Figure 2F)**, suggesting that acquired gefitinib/osimertinib resistance enhances the *in vitro* metastasis potential of NSCLC cells associated with upregulation of MMP family members. HCC827, HCC827/GR and HCC827/OR were then injected subcutaneously into nude mice. Acquired resistance to EGFR-TKIs resulted in a significant increase in the volume of xenograft tumors formed by HCC827 **(Figure 1G-1I and Supplementary Figure 3).** These results suggested Acquired resistance to EGFR-TKIs promotes the tumorigenic function of NSCLC.

### Acquired gefitinib/osimertinib resistance enhances 5'-UTR hypermethylation in NSCLC cells

**Figure 2:**
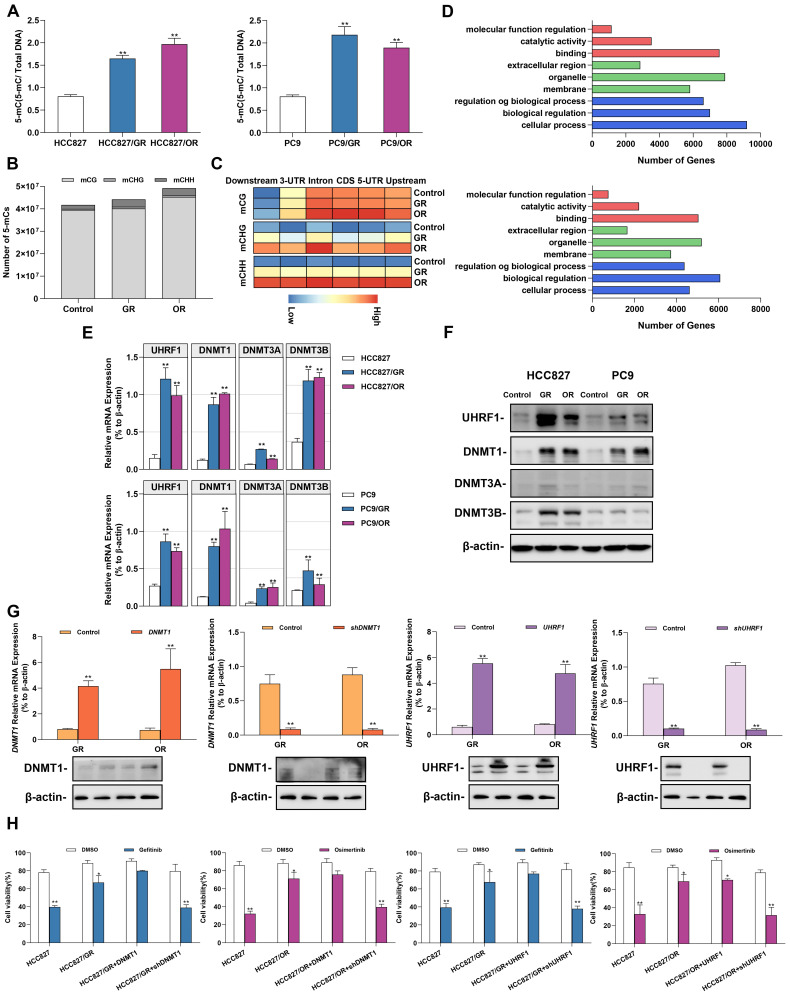
**Alterations in DNA methylation in gefitinib/osimertinib-resistant HCC827 and PC9 cells. (A)** ELISA analysis of global DNA methylation in gefitinib/osimertinib-resistant cells. **(B)** Whole-genome bisulfite sequencing (WGBS) analysis of the DNA methylation number of mCG, mCHG and mCHH sequence contexts.** (C)** The level of DNA methylation (mCG, mCHG and mCHH) profiles across the gene and the flanking regions, including upstream, 5'-untranscript region (5'-UTR), coding sequence (CDS), intron, 3'-untranscript region (3'-UTR) and downstream (-2 kb upstream to +2 kb downstream). (D) KEGG pathway enrichment for the genes overlapping with DMRs in the promoter region of gefitinib/osimertinib-resistant cells. **(E)** and **(F)** RT-qPCR and Western blots analysis of the expression of DNA methyltransferases (*DNMT1*, *DNMT3A* and *DNMT3B*) and *UHRF1* in GR and OR cells. (E) RT-qPCR, (F) RT-qPCR. **(G)***DNMT1* or *UHRF1* was overexpressed or knocked down in HCC827/GR and HCC827/OR. RT-qPCR and Western blotting analysis of DNMT1 and UHRF1. β-actin as sample loading control. **(H)** Cell viability was measured by MTT in gefitinib/osimertinib-resistant cells treatment with gefitinib (1μM) / osimertinib (0.1μM). Data are presented as the mean ± standard deviation (n=3). *p < 0.05, ** *p* < 0.01, significant differences versus control (A and G, one‑way ANOVA with Tukey's post hoc test; H, two‑tailed Student's t‑test).

Environmental stress factors such as acquired drug resistance regulated gene expression by altering epigenetic modifications. We next explored the effect of acquired gefitinib/osimertinib resistance on cellular epigenetic alterations. A global DNA methylation assay showed an increase in the 5-mC ratio in a dose-dependent manner** (Figure 2A and Supplementary Figure 4A)**, suggesting a favorable effect of acquired gefitinib/osimertinib resistance on DNA methylation in NSCLC. We further identified the effect of CpG site methylation on key genes affected in GR/OR cells. Genome-wide DNA methylation profiling revealed that compared to untreated control cells, OR cells exhibited similar proportions of methylation at CpG sites (mCG) but preferentially enhanced DNA methylation at the C site of CHG (mCHG) and CHH (mCHH) in number, while A549/GR increased proportions of methylation at both CG and non-CG sites (mCHG and mCHH) sites **(Figure 2B and Supplementary Figure 5)**. Furthermore, by analysis of the relative amount of DNA methylation changes that occurred along the gene body and flanking regions (2 kb upstream of the TSS and ending 2 kb downstream), the data showed that various and condensed increases in the methylation pattern of mCHG and mCHH contents across genes in GR and OR cells, respectively, whereas the increase in mCG sites was mainly underpinned in the 5'-UTR, including the promoter region** (Figure 2C)**. Moreover, the potential effects of DNA hypermethylation on biological function and related signaling pathways were analyzed using Gene Ontology and KEGG pathway enrichment analysis. As shown in** Figure 2D, Supplementary Figure 4B and 4C, Supplementary Table 6 and Supplementary Table 7**, hypomethylated mCG was mostly enriched in biological regulation activity and signaling pathways involved in tumor regulation.

We further investigated the potential epigenetic pathways associated with acquired gefitinib/osimertinib resistance. We detected the expression of DNA methyltransferases (DNMTs), including DNMT1, DNMT3A and DNMT3B. RT-qPCR and Western blot analysis showed that among the three DNMTs, DNMT1 was most upregulated in GR/OR cells **(Figure 2E and 2F, Supplementary Figure 4D and 4E)**, and was related to the key regulatory protein UHRF1 of DNMTs 40, 41. To demonstrate whether sensitivity to gefitinib/osimertinib is related to the regulation of the UHRF1/DNMT1 complex, we overexpressed or knocked down *DNMT1*/*UHRF1* in GR and OR cells **(Figure 2G and Supplementary Figure 4F)**. The combination of sh*DNMT1* or sh*UHRF1* with EGFR-TKIs significantly reduced proliferation of GR and OR cells compared to treatment with gefitinib or osimotinib alone** (Figure 2H and Figure Supplementary 4G)**. These results suggest that the acquired resistance to gefitinib or osimotinib may be attributed to the regulatory pathway of UHRF1/DNMT1 complex.

### Gefitinib/osimertinib-resistant cells reduced drug sensitivity by epigenetic downregulation of MUC17 expression

**Figure 3:**
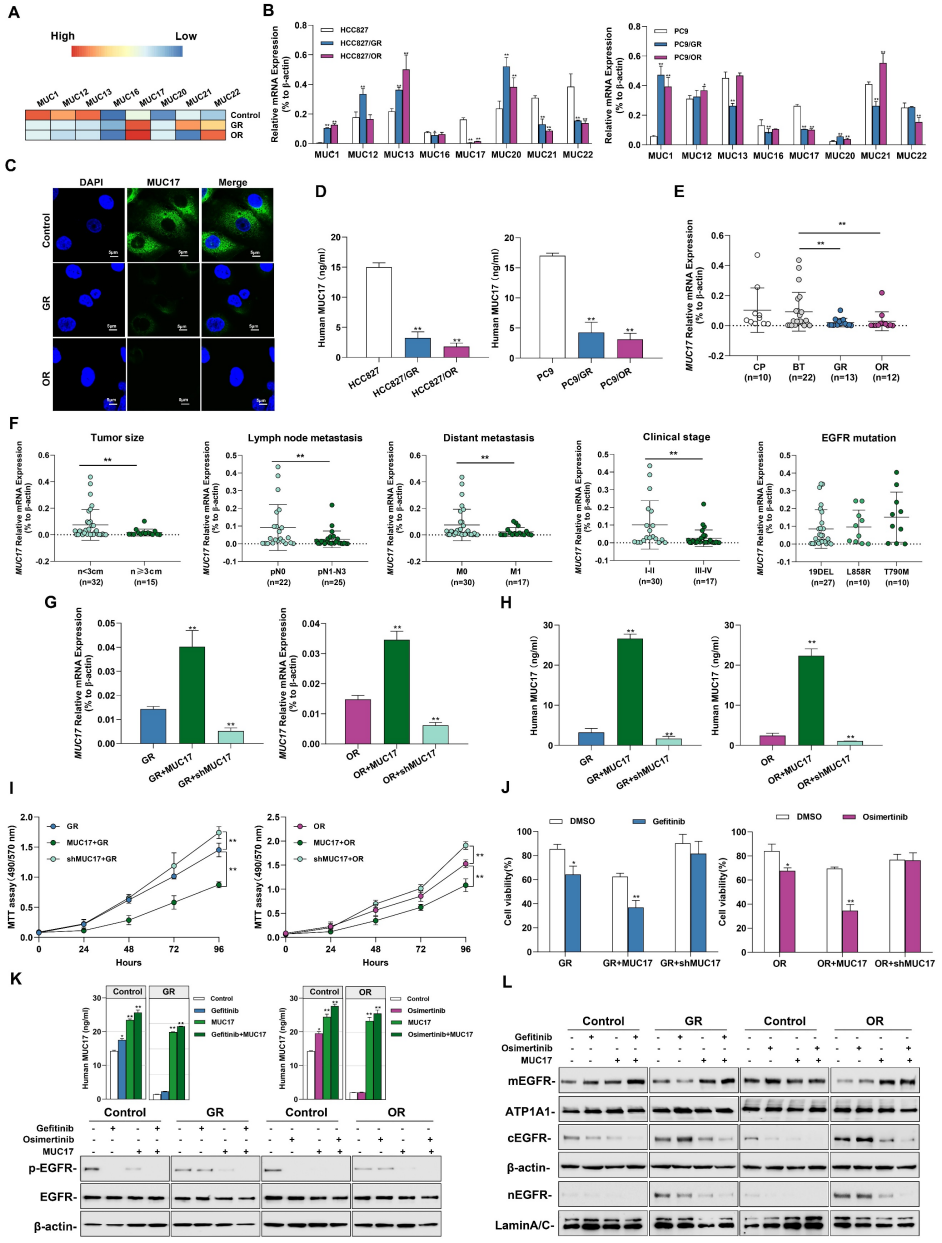
** Gefitinib and osimertinib resistance inhibited MUC17 expression, enhancing lung cancer cell proliferation. (A)** gefitinib/osimertinib resistance-mediated alterations of DNA methylation in the 5'-UTR regions of membrane-bound mucin genes *(MUC1*, *MUC12*, *MUC13*, *MUC16*, *MUC17*, *MUC20*, *MUC21* and *MUC22*).** (B)** RT-qPCR showing the mRNA expression of membrane-bound mucins (*MUCs*) in gefitinib /osimertinib-resistant cells. Each gene was compared with its own untreated control after normalization to β-actin. **(C)** Immunofluorescence staining of MUC17 (green) in HCC827, HCC827/GR and HCC827/OR cells. DAPI-stained nuclei were shown as blue. Scale bar: *5μM.*** (D)** ELISA analysis of MUC17 expression in gefitinib/osimertinib-resistant HCC827 and PC9 cells.** (E)** RT-qPCR of MUC17 expression in BALF from patients with Normal (chronic pneumonia), BT (patient before treatment), GR (gefitinib resistant patient) and OR (osimertinib resistant patient). **(F)** RT-qPCR of *MUC17* expression in BALF from patients with tumor size, lymph node metastasis, distant metastasis, clinical stage and EGFR mutation. **(G)** and **(H)** HCC827/GR and HCC827/OR cells stably expressing ectopic truncated *MUC17* or *shMUC17*. **(G)** The mRNA expression level of *MUC17* was assessed using RT-qPCR. **(H)** The protein expression level of MUC17 was assessed using ELISA. **(I)** MTT showing cell viability. **(J)** MTT showing cell viability treatment with gefitinib (1μM) / osimertinib (0.1μM).** (K)** ELISA analysis of MUC17 and Western blotting analysis of p-EGFR, EGFR in HCC827, HCC827/GR and HCC827/OR were treatment with Gefitinib, Osimertinib and/or transfect with *MUC17*. (L)Western blotting analysis of EGFR in HCC827, HCC827/GR and HCC827/OR were treatment with Gefitinib, Osimertinib and/or transfect with *MUC17*. The protein levels of EGFR subunit are shown in membrane extract(mEGFR), cytoplasmic extracts(cEGFR) and nuclear extracts(nEGFR). ATP1A1 serves as membrane, β-actin as cytoplasmic, and Lamin A/C as nuclear protein loading controls, respectively. Data are presented as the mean ± standard deviation (n=3). One-way ANOVA with Tukey's post-hoc test was performed for B, C, E, G and H, two-tailed Student's t test was used for F. *p < 0.05, **p < 0.01, significant differences from the control.

MUCs constitute the main component of the mucosal barrier and play a central role in maintaining the stability of the internal environment. Membrane-bound mucins act as protectors on epithelial cells and influence the activation of EGFR. Therefore, we measured the expression of membrane-bound mucins in cells. Importantly, we found that acquired resistance to gefitinib/osimertinib resulted in increased methylation of the membrane binding MUCs promoter **(Figure 3A)**. Next, we measured the expression of membrane-bound mucins in GR/OR cells. As shown in **Figure 3B and Supplementary Figure 6A and 6B**, the expression of *MUC17* was significantly reduced in both GR and OR cells. Moreover, the expression of *MUC17* in GR/OR cells decreased in a dose-dependent manner **(Supplementary Figure 6C)**. The results of IF staining and ELISA analysis also confirmed the decreased expression of MUC17 in GR and OR cells **(Figure 3C, 3D, Supplementary Figure 6D and 6E)**. We further verified the expression of *MUC17* in a cohort of bronchoalveolar lavage fluid (BALF) specimens **(Table 1)**, including 10 patients from chronic pneumonia (CP), 22 patients from pre-treatment patients (BT), and 13 patients from gefitinib resistance (GR). 12 patients from osimertinib resistance (OR) **(Supplementary Table 4)**. RT-qPCR analysis showed that *MUC17* expression was significantly reduced in BALF specimens from GR or OR patients compared with those from BT patients **(Figure 3E)**. In addition, we found that the expression of *MUC17* decreased gradually with the increase of tumor malignancy, but was not correlated with EGFR mutation status **(Figure 3F)**. Then, we detected the functional role of MUC17 in GR and OR cells by stably overexpressing the truncated MUC17 (T-MUC17) **(Figure 3G and 3H, Supplementary Figure 6F and 6G)**. The overexpression of MUC17 exerted an anti-proliferation effect and significantly attenuated cell proliferation induced by acquired gefitinib/osimertinib resistance **(Figure 3I and 3J, Supplementary Figure 6H and 6I)**. Furthermore, we also knockdown MUC17 with shRNA in GR and OR cells **(Figure 3G and 3H, Supplementary Figure 6F and 6G)**. In contrast, knockdown of MUC17 led to a significant increase in cell proliferation, further weakening the sensitivity of drug-resistant cells to gefitinib/osimertinib **(Figure 3I and 3J, Supplementary Figure 6H and 6I))**.

To verify the effect of acquired EGFR-TKIs resistance on *MUC17* transcriptional activity, three DNA fragments containing different MUC17-binding motifs (termed FA:-846 bp, FB: -646 bp and FC:-446 bp to the TSS) were cloned and transfected into HCC827 cells^20^, showing that luciferase activity was increased in cells transfected containing FA fragments (-846bp~-646bp, **Supplementary Figure 7A**). Moreover, the results of luciferase reporter assay showed MUC17 promoter region (FA) activity decreased in response to TKI resistance (**Supplementary Figure 7B**). To further explore the correlation between the expression of MUC17 and acquired drug resistance to EGFR-TKIs, we treated GR/OR cells with gefitinib/osimertinib and detected the expression of EGFR-associated proteins. The results showed that gefitinib failed to decrease p-EGFR level inHCC827/GR. Similarly, osimertinib failed to reduce p-EGFR level in HCC827/OR cells. However, the expression of p-EGFR was significantly depleted treatment with gefitinib/osimertinib after overexpression of MUC17 **(Figure 3K)**. To further investigate the effect of MUC17 on EGFR membrane translocation after EGFR-TKIs acquired resistance, we isolated nucleus, cytoplasm and membrane in HCC827, HCC827/GR and HCC827/OR. Overexpression of MUC17 increased the expression of mEGFR while decreased the expression of cEGFR and nEGFR (**Figure 3L).** These results showed that overexpression of MUC17 could increase the sensitivity of cells to EGFR-TKIs by regulating the localization of EGFR in the cell membrane. Given that MUCs are a group of genes presenting epithelial cells as the main protective components of the respiratory mucosal barrier, and their destruction is closely related to changes in the external microenvironment of cells. These findings suggested that the downregulation of MUC17 expression in gefitinib/osimertinib-resistant cells may be associated with 5-UTR hypermethylation.

### Epigenetic downregulation of MUC17 in gefitinib/osimertinib-resistant cells attributable to the UHRF1/DNMT1 complex

**Figure 4:**
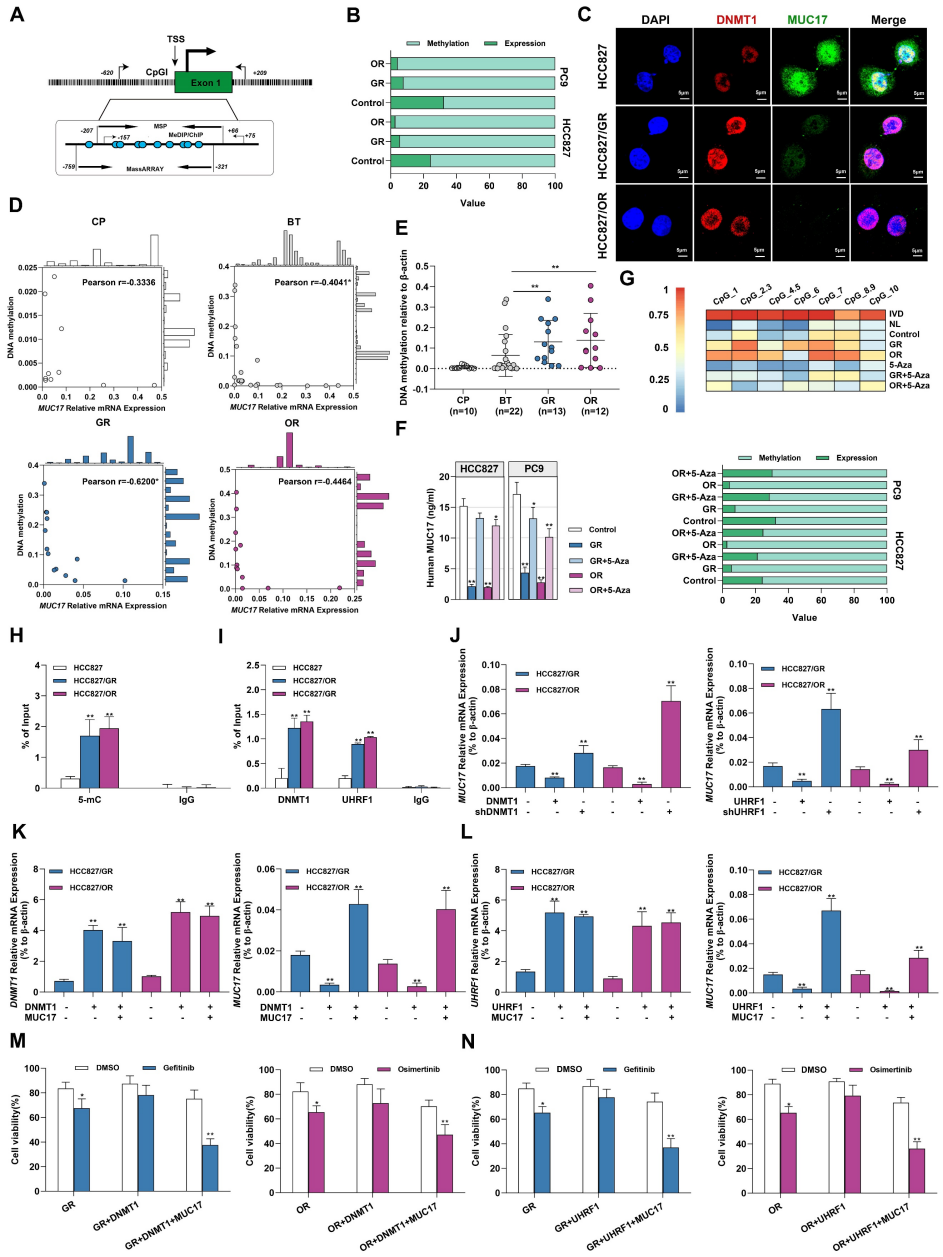
** Gefitinib/osimertinib resistance-mediated epigenetic downregulation of MUC17 through promoter hypermethylation. (A)** Schematic representation of the CpG island around the first exon of *MUC17*. The regions targeted by the primer pairs for MSP-qPCR, ChIP, MeDIP and MassARRAY analysis and their positions relative to the transcription start site (TSS) are shown. **(B)** 100% stacked bar graph showing the association of *MUC17* expression with its promoter methylation in GR and OR cells, RT-qPCR and MS-qPCR were used to detect *MUC17* expression and methylation with the primers listed in **Supplementary Table 1.** The comparison between methylation and expression was visualized by a 100% stacked bar graph. **(C)** Immunofluorescence staining of DNMT1 (red) and MUC17 (green) in HCC827, HCC827/GR and HCC827/OR cells. DAPI-stained nuclei were shown as blue. Scale bar: *5 μM.* **(D)** Pearson correlation analysis of DNA methylation associated with the expression of *MUC17* in BALF specimens**. (E)** MSP-qPCR analysis of DNA methylation in BALF specimens. **(F)** ELISA analysis of MUC17 in HCC827, HCC827/GR and HCC827/OR or treatment with 5-aza-2'-deoxycytidine (5-Aza, 5 μΜ, 96 h). **(G)** MassARRAY and MSP analysis of *MUC17* methylation in HCC827, HCC827/GR and HCC827/OR or treatment with 5-aza-2'-deoxycytidine (5-Aza, 5 μΜ, 96 h). IVD: in vitro methylated DNA served as a positive control; NL: normal blood lymphocyte DNA served as a negative control. **(H)** MeDIP-qPCR analysis of anti-5-mC enriched DNA fragments at the *MUC17* promoter region. The specific enrichment is represented as the percentage of input (% of input) after normalization to the nonspecific IgG control. **(I)** ChIP-qPCR was used to analyze the immunoprecipitated DNA fraction with primers covering the *MUC1*7 promoter region. The normalized enrichment was represented as "% of input". **(J)** RT-qPCR analysis of *MUC17* in HCC827/GR and HCC827/OR stably expressing ectopic *DNMT1* or *shDNMT1*, *UHRF1* or *shUHRF1*.** (K)** RT-qPCR analysis of *DNMT1* and *MUC17* in HCC827/GR and HCC827/OR stably expressing ectopic *DNMT1* and/or *MUC17*.** (L)** RT-qPCR analysis of *UHRF1* and *MUC17* in HCC827/GR and HCC827/OR stably expressing ectopic *UHRF1* and/or *MUC17*.** (M)** and** (N)** MTT showing cell viability treatment with gefitinib (1μM) / osimertinib (0.1μM). Data are presented as the mean ± standard deviation (n=3). One-way ANOVA with Tukey's post-hoc test was performed. *p < 0.05, ** *p* < 0.01, significant differences versus control.

To explore the potential pathways of *MUC17* epigenetic regulation in gefitinib/osimertinib-resistant cells, MS-qPCR and MassARRAY primers covering the *MUC17* promoter region were designed **(Figure 4A)**. Associative analysis of *MUC17* expression and DNA methylation data demonstrated an inverse association between the upstream methylation and mRNA expression of *MUC17*, the methylation of *MUC17* was significantly increased in GR and OR cells **(Figure 4B)**. Moreover, we found a dose-dependent inverse association between *MUC17* expression with its promoter methylation status in GR/OR cells **(Supplementary Figure 8A)**. Further IF staining of HCC827, HCC827/GR and HCC827/OR cells showed that the expression of MUC17 was negatively correlated with the expression of DNMT1 **(Figure 4C)**. To examine the epigenetic contribution of gefitinib/osimertinib acquired resistance to differential expression of *MUC17*, we examined the methylation status in the *MUC17* promoter region of bronchoalveolar fluid (BALF) cohort and calculated whether it was associated with expression. MS-qPCR analysis demonstrated that 61.5% of GR (8/13) and 66.7% of OR (8/12) samples were hypermethylated, whereas the samples from patients with chronic pneumonia (7/10) or BT (16/22) were hypomethylated. Importantly, *MUC17* DNA methylation was highly correlated with expression in GR patients (Pearson r =0.62, P < 0.01) **(Figure 4D)**. Moreover, the data showed that the DNA methylation statues of *MUC17* promoter region was significantly increased in the GR and OR groups compared with the BT group **(Figure 4E and Table 2)**. This reverse association with *MUC17* was partially restored by the DNA methyltransferase inhibitor 5-Aza **(Figure 4F and 4G, Supplementary Figure 8B)**.

We further identified the regions specifically regulating *MUC17* epigenetic silencing in GR/OR cells by MeDIP-qPCR with an anti-5mC antibody. Results showed that GR/OR cells yielded more methylated genomic DNA fragments enriched in the *MUC17* promoter region **(Figure 4H and Supplementary Figure 8C)**. In order to explore the relationship between the hypermethylation of *MUC17* and the regulatory pathway of *UHRF1/DNMT1* complex, we conducted ChIP-qPCR detection. The results showed that anti-DNMT1 or anti-UHRF1 antibodies could enrich more genomic DNA fragments associated with the *MUC17* promoter region in GR/OR cells **(Figure 4I and Supplementary Figure 8D)**. DNMT1 or UHRF1(DNMT1/UHRF1) was overexpressed in GR or OR cells, high expression of DNMT1/UHRF1 inhibited the expression of MUC17. In contrast, knocking down of DNMT1/UHRF1 promoted MUC17 expression and almost reversed gefitinib/osimertinib resistance mediated MUC17 downregulation **(Figure 4J and Supplementary Figure 8E)**. These results suggest that the epigenetic silencing of MUC17 induced by gefitinib/osimertinib resistance is controlled by UHRF1 associated DNMT1. Further, we found that simultaneous overexpression of DNMT1/UHRF1 and MUC17 in OR and GR cells reduced the enhanced effect of DNMT1/UHRF1 on EGFR-TKIs resistance compared with DNMT1 or UHRF1 single-overexpression **(Figure 4K and 4L, Supplementary Figure 8F and 8G)**. In addition, simultaneous overexpression of *DNMT1/UHRF1* and MUC17 restored the sensitivity of drug-resistant cells to gefitinib/osimertinib **(Figure 4M and 4N, Supplementary Figure 8H)**. These results suggested that MUC17 plays a key role in the epigenetic pathway of drug resistance induced by gefitinib/osimertinib mediated by DNMT1/UHRF1 complex.

### The pro-oncogenic effect of gefitinib/osimertinib-resistant cells by downregulation of MUC17 expression via NF-κB activation

**Figure 5:**
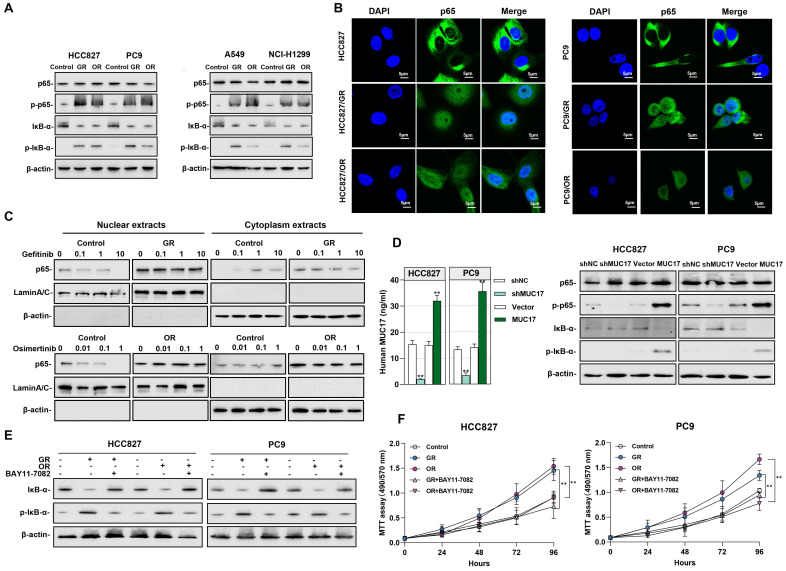
** Gefitinib and osimertinib resistant cells promoted NF-κB activity by inhibiting the expression of MUC17. (A)** Western blotting analysis of p65, p-p65, IκB-α and p-IκB-α expression in gefitinib/osimertinib-resistant cells. **(B)** Immunofluorescence staining of p65 in gefitinib/osimertinib-resistant cells. Scale bars,5*μM*. **(C)** Western blotting analysis of p65 in HCC827, HCC827/GR and HCC827/OR upon gefitinib (0, 0.1, 1, 10μM) or osimertinib (0, 0.01, 0.1, 1μM) treatment. The protein levels of p65 subunit are shown in nuclear extracts and cytoplasmic extracts. β-actin serves as cytoplasmic, and Lamin A/C as nuclear protein loading controls, respectively. **(D)** HCC827 and PC9 were stably expressing ectopic truncated MUC17 or shMUC17. *Left panel:* ELISA analysis of MUC17 in HCC827 and PC9. *Right panel:*Western blotting analysis of p65, p-p65, IκB-α and p-IκB-α expression in HCC827 and PC9.** (E)** and** (F)** Gefitinib and osimertinib resistant cells were treated with BAY11-7082 (8 μM, 12 h). **(E)** Western blotting analysis of IκB-α and p-IκB-α expression in gefitinib and osimertinib resistant cells. β-actin serves as the loading control. **(F)** MTT assays were performed to monitor the viability of cells over 4 days.

We further explored the potential signaling pathways affected by the downregulation of MUC17 in drug-resistant cells, the activation of the NF-κB signaling pathway caused by gefitinib/osimertinib resistance may aggravate inflammation and the progression of malignant tumors. Given the crucial roles in triggering inflammation and malignancy of tumors, we explored NF-κB activity in gefitinib/osimertinib-resistant cells. The results showed that in GR/OR cells, p65 remained unchanged and IκB-α decreased, but phosphorylation of p65 (p-p65) and IκB-α (p-IκB-α) increased **(Figure 5A)**. IF assay suggested that both decreased IκB-α expression and increased p-IκB-α result in the translocation of NF-κB p65 from the cytoplasm into the nucleus to trigger the NF-κB pathway **(Figure 5B)**. We treated drug-resistant cells with EGFR-TKIs and found that gefitinib failed to reduce the level of p65 in the cytoplasm and nucleus of HCC827/GR. The same results were observed in osimertinib-treated OR cells. However, gefitinib/Osimertinib treatment significantly reduced p65 expression in the nucleus and increased in the cytoplasm of sensitive HCC827 cells, suggesting that gefitinib/osimertinib resistance accelerates the progression of malignant tumors through NF-κB activation **(Figure 5C)**. In addition, MUC17 knockdown resulted in an augmentation of p-p65 and p-IκB-α, whereas truncated MUC17 augmented the expression of IκB-α **(Figure 5D)**. Furthermore, the NF-κB inhibitor BAY 11-7082 was shown to attenuate NF-κB activation and cell proliferation induced by gefitinib/osimertinib resistance **(Figure 5E and 5F)**, suggesting that gefitinib/osimertinib-resistant cells promote tumor progression through activation of NF-κB pathway. These results demonstrated that MUC17 plays an important role in the progression of respiratory malignancies induced by gefitinib/osimertinib resistance by inhibiting NF-κB activation.

### UHRF1/DNMT1-modified DNA methylation downregulation MUC17 affected the capacity of MZF1 to regulate IκB-α expression

**Figure 6:**
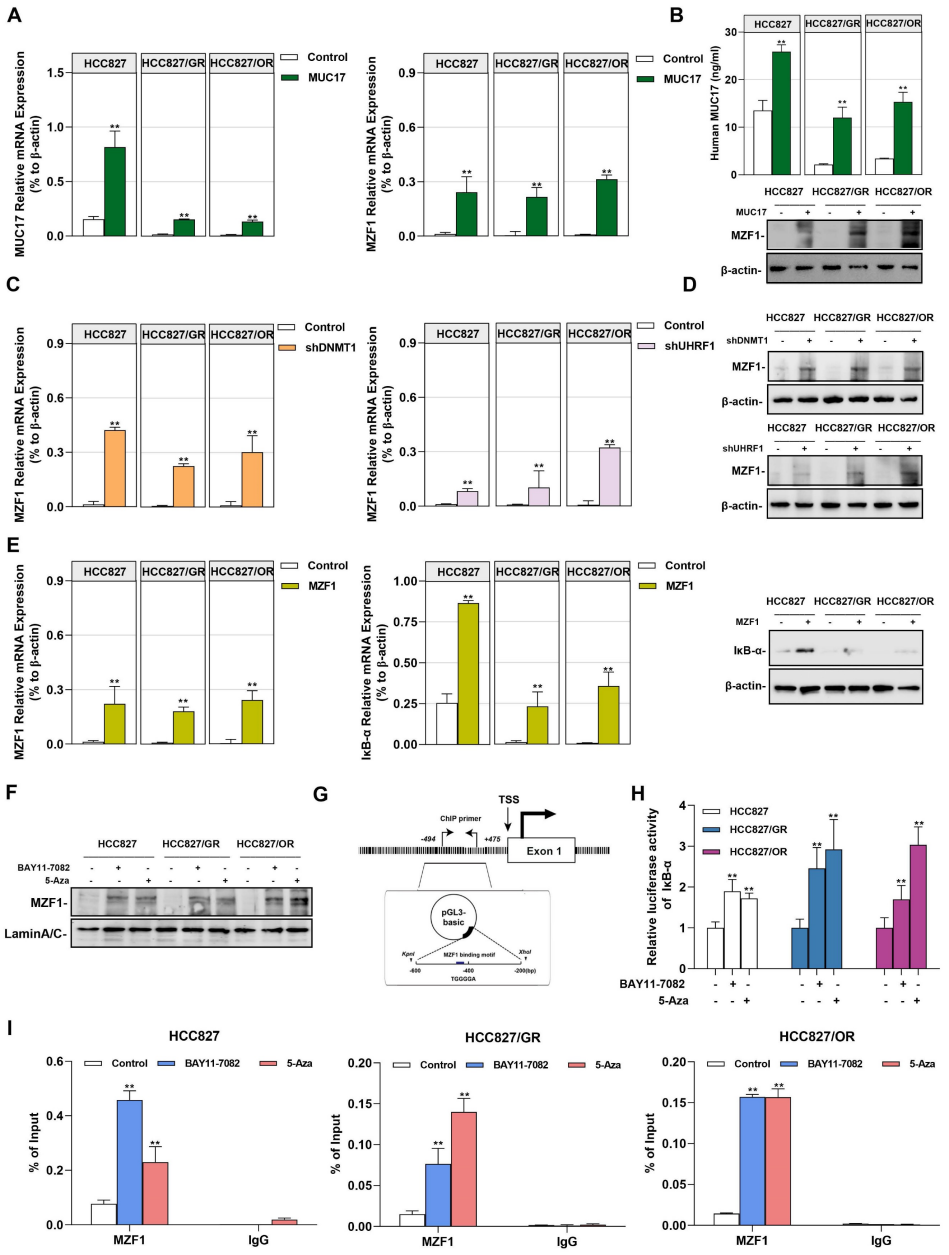
**MZF1 bind to the promoter region of IκB-α regulated by MUC17. (A)** and **(B)** RT-qPCR, ELISA and Western blotting analysis of MZF1 in HCC827, HCC827/GR and HCC827/OR stably expressing ectopic truncated *MUC17*, (A) RT-qPCR, (B) ELISA and Western blotting analysis. **(C)** and **(D)** RT-qPCR and Western blotting analysis of MZF1 in HCC827, HCC827/GR and HCC827/OR stably expressing ectopic *shDNMT1* and *shUHRF1*, (C) RT-qPCR, (D) Western blotting analysis. β-actin as sample loading control. **(E)** RT-qPCR and Western blots analysis of *MZF1* and *IκB-α* in HCC827, HCC827/GR and HCC827/OR stably expressing ectopic *MZF1* or *shMZF1*. *Left panel:* RT-qPCR analysis**.** *Right panel:* Western blots analysis**. (F)** Western blotting analysis of MZF1 in HCC827, HCC827/GR and HCC827/OR treatment with BAY11-7082 or 5-Aza. The protein levels of MZF1 are shown in nuclear extracts. Lamin A/C as nuclear protein loading controls, respectively. **(G)** A schematic illustration of the promoter region of *IκB-α* showing the positions of the consensus sequence of 5'-AGTGGGGA-3' as well as the ChIP-PCR primer set. **(H)** Luciferase reporter assays showing the activity of *IκB-α* promoter region in HCC827, HCC827/GR and HCC827/OR treatment with BAY11-7082 or 5-Aza. **(I)** ChIP-qPCR was used to analyze the immunoprecipitated DNA fraction with primers covering the *IκB-α* promoter region. The normalized enrichment was represented as "% of input". Data are presented as the mean ± standard deviation (n=3). One-way ANOVA with Tukey's post-hoc test was performed. *p < 0.05, ** *p* < 0.01, significant differences versus control.

In our previous studies, we found that MZF1 is regulated by DNA methylation and is an important transcription factor directly promoting the transcription of IκB-α 36. Considering that zinc finger proteins preferentially bind to the GC-rich motif, we hypothesized that MZF1 is a methyl-sensitive transcription factor involved in epigenetic methylation of NF-κB regulated by UHRF1/DNMT1. Therefore, we investigated whether MUC17 inhibited NF-κB activation through MZF1. To verify this hypothesis, we first examined the expression of MZF1 in gefitinib/osimertinib-resistant cells, we found that MZF1 was weakly expressed in the protein level, but a decreased in mRNA level of MZF1 was observed in EGF-TKIs resistant cell lines **(Supplementary Figure 9)**. We further explored the relationship between MUC17 and MZF1. MUC17 overexpression resulted in the increased expression of MZF1, but overexpression of MZF1 had no effect on MUC17 expression in HCC827, HCC827/GR and HCC827/OR, suggesting that MUC17 regulates the expression of MZF1 **(Figure 6A, 6B and Supplementary Figure 10)**. Moreover, p-EGFR expression was significantly reduced in MZF1 overexpressed GR/OR cells treated with gefitinib/osititinib. The results suggested that MUC17 regulated EGFR activity by promoting the expression of MZF1 (**Supplementary Figure 11).** Further, we explored the regulation of MZF1 expression by the UHRF1/DNMT1 complex, and found that the UHRF1/DNMT1 complex inhibited MZF1 expression **(Figure 6C and 6D)**. The results showed that UHRF1/ DNMT1 inhibited the expression of MZF1 by downregulating the expression of MUC17. To investigate the relationship between MZF1 and NF-κB activation, we determined the expression of IκB-α in NSCLC cells transfected with MZF1 vector. We found that MZF1 enhanced the expression of IκB-α at mRNA and protein levels **(Figure 6E)**. Meanwhile, both NF-κB signaling inhibitors BAY11-7082 and methylation enzyme inhibitors 5-Aza enhanced nuclear translocation of MZF1 **(Figure 6F)**. To verify the specific binding of MZF1 to IκB-α, we cloned DNA fragments containing MZF1-binding motifs (-494~-212bp from TSS) and transfected them into HCC827, HCC827/GR, and HCC827/OR cells **(Figure 6G)**. The results showed that the transfected cells treatment with BAY11-7082 or 5-Aza displayed significantly increased luciferase activity compared with that of the control **(Figure 6H)**. Chip-qpcr analysis showed an increase in anti-MZF1 antibody enriched DNA fragments in BAY11-7082 or 5-Aza-treated cells, indicating specific binding of MZF1 to IκB-α **(Figure 6I)**.

**Figure 7:**
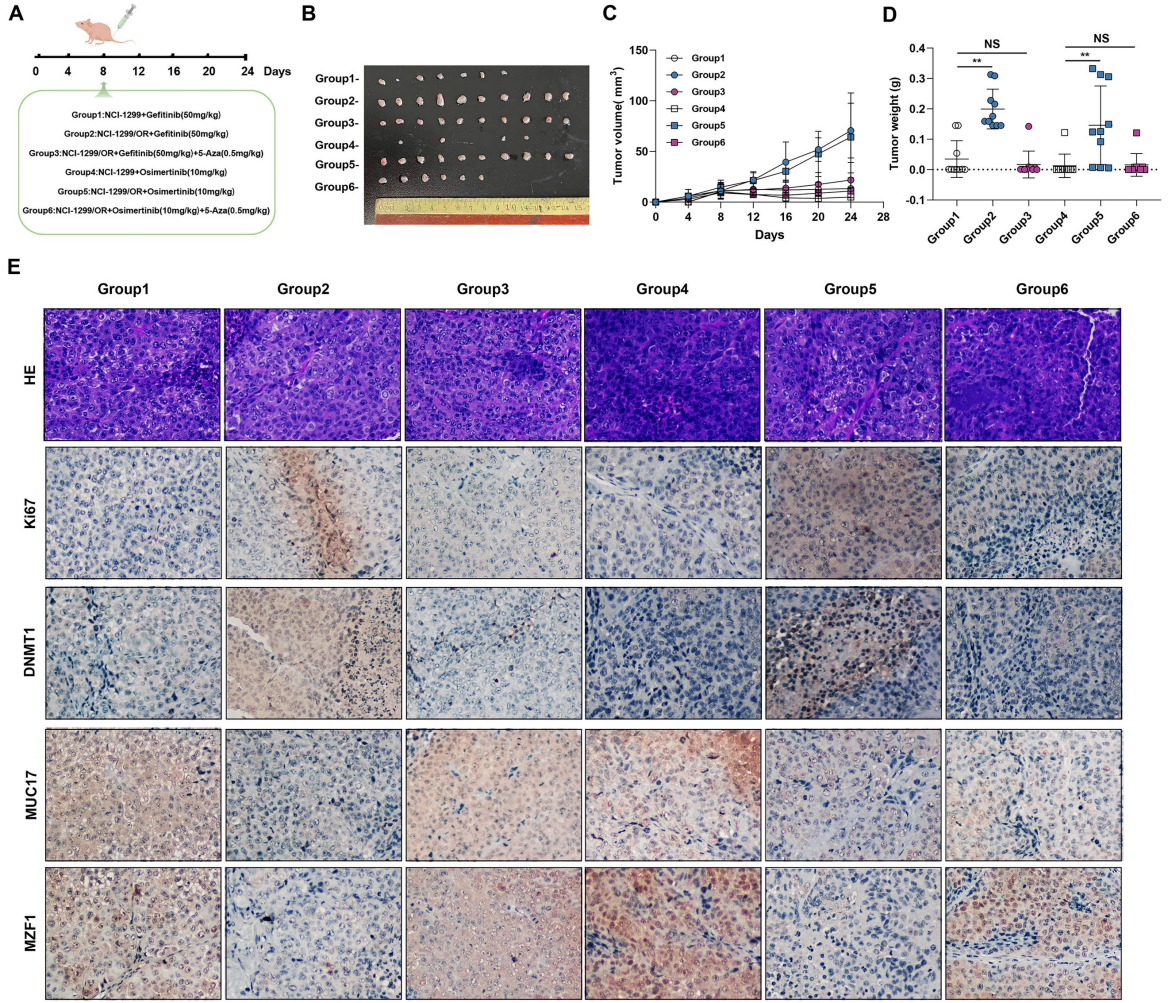
**DNMT1 inhibitor enhance the sensitivity of EGR-TKIs. (A)** NCI-H1299, NCI-H1299/GR and NCI-H1299/OR cells were injected into the left and right flanks of nude mice (n = 2 flanks × 5 mice in each group). On the day 8, mice were treated with Gefitinib and/or 5-Aza, Osimertinib and/or 5-Aza. **(B)** Photographs of xenograft tumors from nude mice on day 24. **(C)** Tumor volumes of tumor-bearing mice measured at the indicated time points. **(D)** Tumor weights of the mice xenografts. **(E)** H&E and Immunohistochemical staining of Ki67, DNMT1, MUC17 and MZF1 in isolated xenograft tumors. Brown staining indicates the expression of protein. Each image is shown at a magnification of 200×. Data are presented as the mean ± standard deviation (n=3). One-way ANOVA with Tukey's post-hoc test was performed. *p < 0.05, ** *p* < 0.01, significant differences versus control.

We further verified this *in vivo* by injecting gefitinib/osimertinib-resistant cells cells into the subcutaneously of nude mice treated with EGFR-TKIs and /or 5-Aza **(Figure 7A)**. The *in vivo* lung tumorigenic assay showed that a significant increase in xenograft tumor volume formed by gefitinib/osimertinib-resistant cells in nude mice with gefitinib/osimertinib treatment. Conversely, the EGFR-TKIs and 5-Aza combination groups showed a significant reduction in the volume of xenograft tumors formed by gefitinib/osimertinib-resistant cells **(Figure 7B-7D, Supplementary Figure 12)**. These results indicate that EGFR-TKIs+5-Aza possessed enhanced anti-cancer activity with fewer side effects in mice. Further IHC analysis of the xenograft tumor sections showed that MZF1 expression was higher after EGFR-TKIs+5-Aza treatment compared with EGFR-TKIs alone. This was associated with upregulation of MUC17, but downregulation of Ki67 and DNMT1 **(Figure 7E)**. Thus, the combination of two agents demonstrates an enhanced effect of anti-cancer activity in which 5-Aza-induced upregulations MUC17 and MZF1 may promote the sensitivity of NSCLC resistant cells to EGFR-TKIs.

Taken together, our results revealed an epigenetic pathway responsible for the regulation of MUC17 expression that was controlled by UHRF1/DNMT1-mediated DNA hypermethylation, and that the dysregulation of this pathway may cause EGFR-TKIs resistance.

## Discussion

Treatment with tyrosine kinase inhibitors (TKIs) has brought significant benefits to lung cancer patients with EGFR mutations 42. Although the therapeutic effects of TKIs have improved the prognosis of NSCLC patients, patients are highly likely to develop resistance to TKIs 43. Therefore, it is necessary to develop new biomarkers to monitor TKIs resistance. In this study, we found that the statutes of methylation in drug-resistant cells induced by different concentrations of gefitinib/osimertinib was different in a dose-dependent manner. Malignant proliferation of tumor cells induced by gefitinib/osimertinib resistance were due to hypermethylation of the MUC17-specific promoter caused by the UHRF1/DNMT1 complex, which activates the NF-κB signaling pathway. Therefore, MUC17 plays an inhibitory role in lung malignant tumors and is a biomarker for monitoring EGFR-TKIs resistance.

Solving the acquired resistance of EGFR-TKIs has been the focus and difficulty of research in recent years, most of which focused on the influence of gene copy number changes on drug resistance 44, 45. EGFR-TKIs resistance have recently been found to induce epigenetic alterations in cells, including methylation and acetylation 46-49. Studies have also shown that EGFR-TKI resistance can cause functional changes of tumor cells and DNA hypermethylation of tumor suppressor gene (TSG), and epigenetic intervention might be an effective strategy to reverse EGFR-TKI resistance 48. Moreover, DNA methylation could be a novel tumor cellular state, which helps to accurately distinguish the tumor heterogeneity of EGFR-Tkis. Epigenome factors may complement DNA mutation status to identify patients with lung adenocarcinoma who are unlikely to benefit from EGFR-TKI therapy 47.

Our results showed that gefitinib/osimertinib resistance increased proliferation and metastasis of lung cancer cells. Further analysis by WGBS showed that this process resulted in DNA hypermethylation and significantly induced 5-mC expression. The expression of tumor suppressor genes (TSGs) is epigenetic regulated by genomic DNA methylation and histone modification patterns. Abnormal modifications have been associated with cancer development, including CpG island methylation of promoters or intragenic regions 50. Our findings demonstrated that acquired gefitinib/osimertinib resistance enhances methylation levels in mCG promoters and intragenic regions. However, the promoter regions (upstream of TSS) were relatively higher than the gene body. In addition, methylated mCG induced by gefitinib/osimertinib resistance is mainly enriched in signaling pathways, such as transcriptional misregulation in cancer, indicating that acquired gefitinib/osimertinib resistance may cause TSG silencing through promoter epigenetic alteration, thus leading to drug-resistance-induced tumor development.

MUCs constitute the main component of mucosal barrier and play a central role in maintaining internal environmental stability. Mucins are classified into membrane-bound and secretory types according to their expression patterns 15, 51, in which membrane-bound mucins act as protectors on epithelial cells. Disruption of MUCs expression has been reported in several cancer types, including lung cancer 22, 51, 52. In addition, several well-known tumor-associated antigens on mucins serve as biomarkers for cancer, especially adenocarcinoma 59,60. As a membrane-bound mucin, MUC17 plays a major protective role by limiting the adhesion of harmful substances to epithelial cell surfaces 51, 53, 54. Epigenetic regulation of mucin expression, such as MUC1, MUC2, MUC4, MUC5AC and MUC17, has been reported to be controlled by DNA methylation in a variety of cancer cells 21. However, members of the transmembrane mucins play opposite roles in drug resistance and tumor progression. Studies have shown that MUCs has a regulatory effect on EGFR 55, 56. The SEA domain of MUCs in drug-resistant cells were shed under the action of matrix metalloproteinase-7 (MMP-7), releasing MUC1-C-ter and promoting the nucleolus transport of p53 in gefitinib-resistant cells 55. Here, we demonstrated a more complicated epigenetic regulation of MUC17 in lung cancer. Our results showed that gefitinib/osimertinib resistance resulted in genome-wide hypermethylation and decreased expression of MUC17. We found that the downregulation of MUC17 expression in gefitinib/osimertinib-resistant cells was due to the DNA hypermethylation of the MUC17 promoter, suggesting that DNA methylation was closely related to the downregulation of MUC17. We further investigated the potential epigenetic pathways associated with acquired gefitinib/osimertinib resistance. UHRF1, a key regulatory protein of DNA methylation, is thought to bind to H3K9me2/3 or semi-methylated CpG and recruit DNMT1 to the DNA replication fork to maintain DNA methylation 57, 58. Our study found that UHRF1/DNMT1 complex bind to the promoter region of MUC17 and increased DNA methylation modification, suggesting that DNA methylation modification in the promoter region of MUC17 may be a potential marker of acquired drug resistance to EGFR-TKIs.

Activation of the NF-κB pathway has been shown to be closely associated with acquired resistance to EGFR-TKIs 24, 25. Blocking NF-κB activation by increasing the expression of IκB-α in cancer cells has been shown to have good anti-tumor effects. IκB-α has been identified as a potential target for inhibiting NF-κB activation 59. We observed that the expression of IκB-α was decreased, but there was an increase in p-IκB-α expression. The expression of p65 decreased in the cytoplasm but was enhanced in the nucleus. Abnormal expression of MUC17 regulates the activation of NF-κB pathway 20, 22. Our results showed that MUC17 positively regulates the expression of IκB-α. However, MUC17 as a membrane protein, is unlikely to regulate IκB-α activity by directly binding to its promoter. We previously found that MZF1 directly promotes transcription of IκB-α 36. Studies have shown that zinc activate EGFR by activating intracellular Src and metalloproteinases. Activated EGFR induces respiratory inflammation by up regulating the expression of inflammatory proteins. Members of the zinc finger protein family play an important role in regulating the expression and activity of EGFR 60-62. The gain- and loss- of function assays in this study further demonstrated the regulatory capacity of MUC17 in control of MZF1 expression. Therefore, we used MZF1 overexpressing cell lines to investigate the inhibitory effect of MZF1 on phosphorylation of EGFR. The results demonstrate that the high expression of MZF1 may inhibit EGFR phosphorylation by promoting binding to zinc ions. The results showed that UHRF1/DNMT1-mediated down-regulation of MUC17 reduced MZF1 nuclear translocation, and MZF1 directly bound to its promoter to activate IκB-α transcription. These findings suggest that MUC17 restored drug sensitivity in gefitinib/osimertinib-resistant cells by inhibiting the NF-κB pathway.

## Conclusion

**Figure 8:**
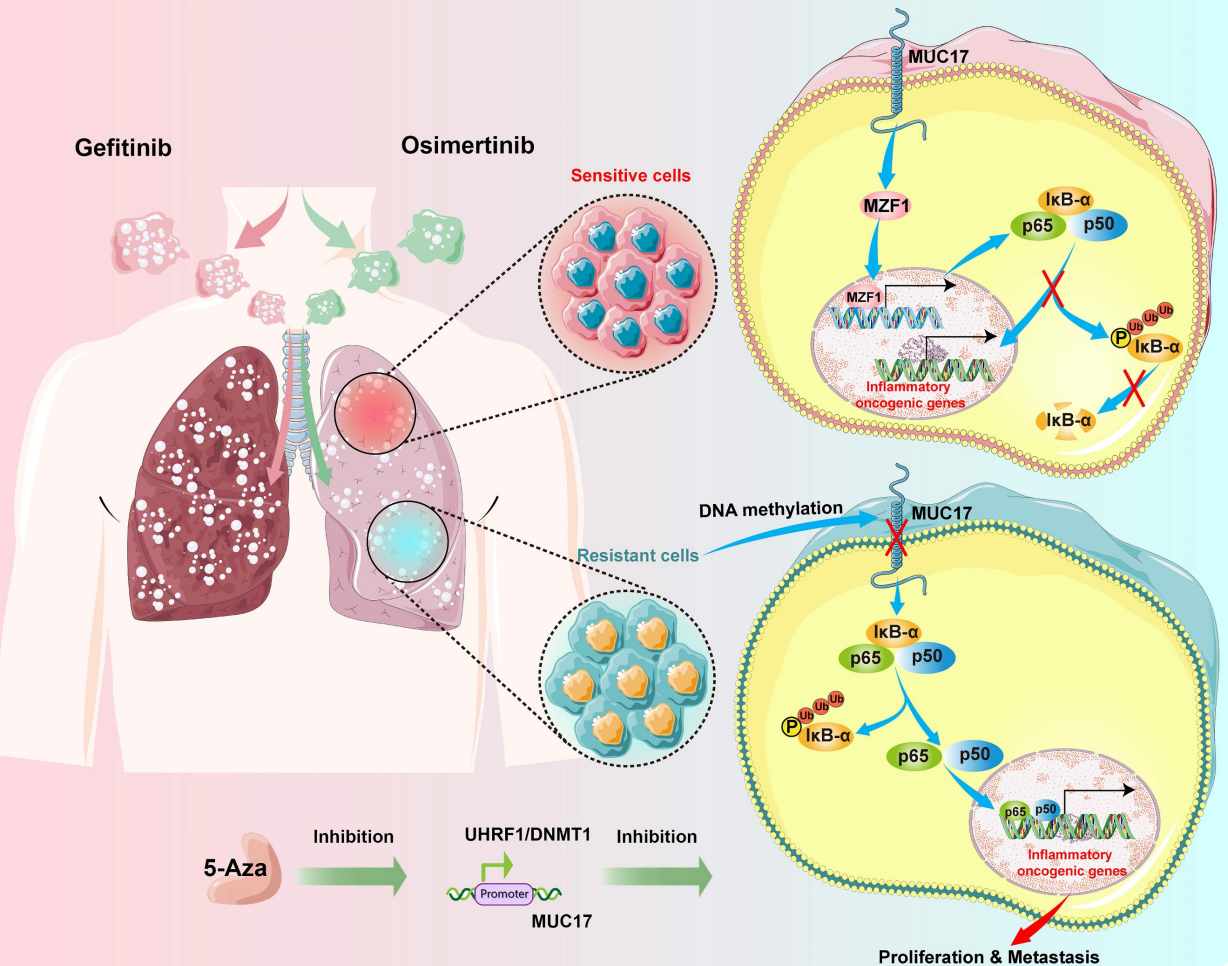
Schematic illustration of the molecular mechanism of MUC17 in drug-resistant cells.

In summary, our study reveals that long-term gefitinib/osimertinib exposure enhances genome-wide methylation and promotes drug resistance in lung cancer cells through UHRF1/DNMT1-mediated epigenetic silencing of MUC17. Our results suggest that MUC17 promotes drug sensitivity by upregulating MZF1 expression and inhibiting NF-κB activity in gefitinib/osimertinib -resistant cells. Therefore, MUC17 may serve as an epigenetic sensor for biological monitoring of resistance to EGFR-TKIs (**Figure 8**).

## Supplementary Material

Supplementary figures and tables.

## Figures and Tables

**Table 1:** The association of MUC17 expression with clinicpathologic parameters in NSCLC

Characteristics	Cases	MUC17 expression		
		High (%)	Low (%)	*P*-value
Total	57	16 (28.1)	41 (71.9)	
**Gender**				
Male	26	7 (26.9)	19 (73.1)	
Female	31	9 (29.0)	22 (71.0)	0.8599
**Age at diagnosis**				
≥60	39	11(28.2)	28 (71.8)	
<60	18	5 (27.8)	13 (72.2)	0.9733
**Pathological type**				
Chronic pneumonia	10	4 (40.0)	6 (60.0)	
Lung adenocarcinoma	43	11 (25.6)	32 (74.4)	0.3619
Lung squamous cell carcinomas	4	1 (25.0)	3 (75.0)	0.5967
**Tumor invasive depth**				
NA	10			
1	18	7 (38.9)	11 (61.1)	
2	14	4 (28.6)	10 (71.4)	**0.027**
3	8	0 (0.0)	8 (100.0)	**0.0391**
4	7	1 (14.3)	6 (85.7)	0.2364
**Lymph node metastasis**				
NA	10			
0	22	9 (40.9)	13 (59.1)	
1	2	0 (0.0)	2 (100.0)	0.2526
2	12	2 (16.7)	10 (83.3)	0.1488
3	11	1 (9.0)	10 (91.0)	0.0608
**Distant metastasis**				
NA	10			
Yes	17	3 (17.6)	14 (82.4)	
No	30	9 (30.0)	21 (70.0)	0.3507
**Clinic stage**				
NA	10			
I	18	8 (44.4)	10 (55.6)	
II	1	0 (0.0)	1 (100.0)	0.3809
III	9	1 (11.1)	8 (88.9)	0.0832
IV	19	3 (15.8)	16 (84.2)	**0.0566**
**EGFR mutation**				
NA	10			
19DEL	27	10 (37.0)	17 (63.0)	
L858R	10	5 (50.0)	5 (50.0)	0.4757
T790M	10	6 (60.0)	4 (40.0)	0.2105

**Table 2:** The association of MUC17 methylation with clinicopathologic parameters in NSCLC

Characteristics	Cases	MUC17 Methylation		
		High (%)	Low (%)	*P*-value
Total	57	21 (36.8)	36 (63.2)	
**Gender**				
Male	26	13 (50.0)	13 (50.0)	
Female	31	8 (25.8)	23 (74.2)	0.1019
**Age at diagnosis**				
≥60	39	14(35.9)	25 (64.1)	
<60	18	7 (38.9)	13 (61.1)	0.9457
**Pathological type**				
Chronic pneumonia	10	0 (0.0)	10 (100.0)	
Lung adenocarcinoma	43	20 (46.5)	23 (53.5)	**0.0063**
Lung squamous cell carcinomas	4	1 (25.0)	3 (75.0)	0.1008
**Tumor invasive depth**				
NA	10			
1	18	5 (27.8)	13 (72.2)	
2	14	8 (57.1)	6 (42.9)	**0.0049**
3	8	5 (62.5)	3 (37.5)	**0.0336**
4	7	3 (42.9)	4 (57.1)	0.3905
**Lymph node metastasis**				
NA	10			
0	22	6 (27.3)	16 (72.7)	
1	2	1 (50.0)	1 (50.0)	0.6685
2	12	8 (66.7)	4 (33.3)	**0.0005**
3	11	6 (54.5)	5 (45.5)	**0.0228**
**Distant metastasis**				
NA	10			
Yes	17	8 (47.1)	9 (52.9)	
No	30	13 (43.3)	17 (56.7)	0.5454
**Clinic stage**				
NA	10			
I	18	5 (27.8)	13 (72.2)	
II	1	0 (0.0)	1 (100.0)	0.7836
III	9	7 (77.8)	2 (22.2)	**0.0009**
IV	19	9 (47.4)	10 (52.6)	**0.0167**

## Abbreviations

5-Aza: 5-aza-2'-deoxycytidine
BALF: Bronchoalveolar lavage fluid
ChIP: Chromatin immunoprecipitation
DNMT1: DNA methyltransferase 1
EGFR: Epidermal growth factor receptor
ELISA: Enzyme-linked immunosorbent assay
GR: Gefitinib-resistant cells
IF: Immunofluorescence staining
IHC: Immunohistochemistry
MeDIP-qPCR: Methylated DNA immunoprecipitation-qPCR
MS-qPCR: Methylation-specific qPCR
MUC17: Mucin 17, cell surface associated
MZF1: Methyl-sensitive myeloid zinc finger 1
NSCLC: Non-small-cell lung cancer
OR: Osimertinib-resistant cells
TKIs: Tyrosine kinase inhibitors
UHRF1: Ubiquitin-like with PHD and RING finger domain 1

## References

[1] Sung H, Ferlay J, Siegel RL, Laversanne M, Soerjomataram I, Jemal A, Bray F (2021). Global Cancer Statistics 2020: GLOBOCAN Estimates of Incidence and Mortality Worldwide for 36 Cancers in 185 Countries. *CA Cancer J Clin*.

[2] Chen W, Zheng R, Baade PD, Zhang S, Zeng H, Bray F, Jemal A, Yu XQ, He J (2016). Cancer statistics in China, 2015. *CA Cancer J Clin*.

[3] Cooper AJ, Sequist LV, Lin JJ (2022). Third-generation EGFR and ALK inhibitors: mechanisms of resistance and management. *Nat Rev Clin Oncol*.

[4] Thress KS, Paweletz CP, Felip E, Cho BC, Stetson D, Dougherty B, Lai Z, Markovets A, Vivancos A, Kuang Y (2015). Acquired EGFR C797S mutation mediates resistance to AZD9291 in non-small cell lung cancer harboring EGFR T790M. *Nat Med*.

[5] Ercan D, Choi HG, Yun CH, Capelletti M, Xie T, Eck MJ, Gray NS, Jänne PA (2015). EGFR Mutations and Resistance to Irreversible Pyrimidine-Based EGFR Inhibitors. *Clin Cancer Res*.

[6] Yu HA, Tian SK, Drilon AE, Borsu L, Riely GJ, Arcila ME, Ladanyi M (2015). Acquired Resistance of EGFR-Mutant Lung Cancer to a T790M-Specific EGFR Inhibitor: Emergence of a Third Mutation (C797S) in the EGFR Tyrosine Kinase Domain. *JAMA Oncol*.

[7] Schoenfeld AJ, Chan JM, Kubota D, Sato H, Rizvi H, Daneshbod Y, Chang JC, Paik PK, Offin M, Arcila ME (2020). Tumor Analyses Reveal Squamous Transformation and Off-Target Alterations As Early Resistance Mechanisms to First-line Osimertinib in EGFR-Mutant Lung Cancer. *Clin Cancer Res*.

[8] Pailler E, Faugeroux V, Oulhen M, Mezquita L, Laporte M, Honoré A, Lecluse Y, Queffelec P, NgoCamus M, Nicotra C (2019). Acquired Resistance Mutations to ALK Inhibitors Identified by Single Circulating Tumor Cell Sequencing in ALK-Rearranged Non-Small-Cell Lung Cancer. *Clin Cancer Res*.

[9] Eberlein CA, Stetson D, Markovets AA, Al-Kadhimi KJ, Lai Z, Fisher PR, Meador CB, Spitzler P, Ichihara E, Ross SJ (2015). Acquired Resistance to the Mutant-Selective EGFR Inhibitor AZD9291 Is Associated with Increased Dependence on RAS Signaling in Preclinical Models. *Cancer Res*.

[10] Crystal AS, Shaw AT, Sequist LV, Friboulet L, Niederst MJ, Lockerman EL, Frias RL, Gainor JF, Amzallag A, Greninger P (2014). Patient-derived models of acquired resistance can identify effective drug combinations for cancer. *Science*.

[11] Chen Z, Chen Q, Cheng Z, Gu J, Feng W, Lei T, Huang J, Pu J, Chen X, Wang Z (2020). Long non-coding RNA CASC9 promotes gefitinib resistance in NSCLC by epigenetic repression of DUSP1. *Cell Death Dis*.

[12] Raoof S, Mulford IJ, Frisco-Cabanos H, Nangia V, Timonina D, Labrot E, Hafeez N, Bilton SJ, Drier Y, Ji F (2019). Targeting FGFR overcomes EMT-mediated resistance in EGFR mutant non-small cell lung cancer. *Oncogene*.

[13] Avram GE, Marcu A, Moatar A, Samoila C, Podariu A, Seclaman E, Marian C (2020). Changes in global DNA methylation and hydroxymethylation in oral mucosa according to tobacco smoke exposure. *J Int Med Res*.

[14] Cheng FJ, Chen CH, Tsai WC, Wang BW, Yu MC, Hsia TC, Wei YL, Hsiao YC, Hu DW, Ho CY (2021). Cigarette smoke-induced LKB1/AMPK pathway deficiency reduces EGFR TKI sensitivity in NSCLC. *Oncogene*.

[15] Hollingsworth MA, Swanson BJ (2004). Mucins in cancer: protection and control of the cell surface. *Nat Rev Cancer*.

[16] Senapati S, Sharma P, Bafna S, Roy HK, Batra SK (2008). The MUC gene family: their role in the diagnosis and prognosis of gastric cancer. *Histology & Histopathology*.

[17] Yang T, Sim KY, Ko GH, Ahn JS, Kim HJ, Park SG (2022). FAM167A is a key molecule to induce BCR-ABL-independent TKI resistance in CML via noncanonical NF-κB signaling activation. *J Exp Clin Cancer Res*.

[18] Takahashi S, Noro R, Seike M, Zeng C, Matsumoto M, Yoshikawa A, Nakamichi S, Sugano T, Hirao M, Matsuda K (2021). Long Non-Coding RNA CRNDE Is Involved in Resistance to EGFR Tyrosine Kinase Inhibitor in EGFR-Mutant Lung Cancer via eIF4A3/MUC1/EGFR Signaling. *Int J Mol Sci*.

[19] Luo Y, Ma S, Sun Y, Peng S, Zeng Z, Han L, Li S, Sun W, Xu J, Tian X (2021). MUC3A induces PD-L1 and reduces tyrosine kinase inhibitors effects in EGFR-mutant non-small cell lung cancer. *Int J Biol Sci*.

[20] Yang B, Wu A, Hu Y, Tao C, Wang JM, Lu Y, Xing R (2019). Mucin 17 inhibits the progression of human gastric cancer by limiting inflammatory responses through a MYH9-p53-RhoA regulatory feedback loop. *J Exp Clin Cancer Res*.

[21] Chen H, Yang X, Liu H, Ma K, Zhong J, Dong Z, Zhuo M, Wang Y, Li J, An T (2017). Correlation between Serum Tumor Markers and Efficacy of First-line EGFR-TKIs in Patients with Advanced Lung Adenocarcinoma. *Zhongguo Fei Ai Za Zhi*.

[22] Lin S, Zhang Y, Hu Y, Yang B, Cui J, Huang J, Wang JM, Xing R, Lu Y (2019). Epigenetic downregulation of MUC17 by H. pylori infection facilitates NF-κB-mediated expression of CEACAM1-3S in human gastric cancer. *Gastric Cancer*.

[23] Lin S, Tian C, Li J, Liu B, Ma T, Chen K, Gong W, Wang JM, Huang J (2021). Differential MUC22 expression by epigenetic alterations in human lung squamous cell carcinoma and adenocarcinoma. *Oncol Rep*.

[24] Li L, Wang T, Hu M, Zhang Y, Chen H, Xu L (2020). Metformin Overcomes Acquired Resistance to EGFR TKIs in EGFR-Mutant Lung Cancer via AMPK/ERK/NF-κB Signaling Pathway. *Front Oncol*.

[25] Chiu CF, Chang YW, Kuo KT, Shen YS, Liu CY, Yu YH, Cheng CC, Lee KY, Chen FC, Hsu MK (2016). NF-κB-driven suppression of FOXO3a contributes to EGFR mutation-independent gefitinib resistance. *Proc Natl Acad Sci U S A*.

[26] Wang X, Yin H, Zhang H, Hu J, Lu H, Li C, Cao M, Yan S, Cai L (2018). NF-κB-driven improvement of EHD1 contributes to erlotinib resistance in EGFR-mutant lung cancers. *Cell Death Dis*.

[27] Nygaard M, Terkelsen T, Vidas Olsen A, Sora V, Salamanca Viloria J, Rizza F, Bergstrand-Poulsen S, Di Marco M, Vistesen M, Tiberti M (2016). The Mutational Landscape of the Oncogenic MZF1 SCAN Domain in Cancer. *Front Mol Biosci*.

[28] Eguchi T, Prince T, Wegiel B, Calderwood SK (2015). Role and Regulation of Myeloid Zinc Finger Protein 1 in Cancer. *J Cell Biochem*.

[29] Hromas R, Davis B, Rauscher FJ 3rd, Klemsz M, Tenen D, Hoffman S, Xu D, Morris JF (1996). Hematopoietic transcriptional regulation by the myeloid zinc finger gene, MZF-1. *Curr Top Microbiol Immunol*.

[30] Hromas R, Morris J, Cornetta K, Berebitsky D, Davidson A, Sha M, Sledge G, Rauscher F 3rd (1995). Aberrant expression of the myeloid zinc finger gene, MZF-1, is oncogenic. *Cancer Res*.

[31] Hromas R, Boswell S, Shen RN, Burgess G, Davidson A, Cornetta K, Sutton J, Robertson K (1996). Forced over-expression of the myeloid zinc finger gene MZF-1 inhibits apoptosis and promotes oncogenesis in interleukin-3-dependent FDCP.1 cells. *Leukemia*.

[32] Deng Y, Wang J, Wang G, Jin Y, Luo X, Xia X, Gong J, Hu J (2013). p55PIK transcriptionally activated by MZF1 promotes colorectal cancer cell proliferation. *Biomed Res Int*.

[33] Peterson FC, Hayes PL, Waltner JK, Heisner AK, Jensen DR, Sander TL, Volkman BF (2006). Structure of the SCAN domain from the tumor suppressor protein MZF1. *J Mol Biol*.

[34] Mudduluru G, Vajkoczy P, Allgayer H (2010). Myeloid zinc finger 1 induces migration, invasion, and in vivo metastasis through Axl gene expression in solid cancer. *Mol Cancer Res*.

[35] Vishwamitra D, Curry CV, Alkan S, Song YH, Gallick GE, Kaseb AO, Shi P, Amin HM (2015). The transcription factors Ik-1 and MZF1 downregulate IGF-IR expression in NPM-ALK(+) T-cell lymphoma. *Mol Cancer*.

[36] Lin S, Wang X, Pan Y, Tian R, Lin B, Jiang G, Chen K, He Y, Zhang L, Zhai W (2019). Transcription Factor Myeloid Zinc-Finger 1 Suppresses Human Gastric Carcinogenesis by Interacting with Metallothionein 2A. *Clin Cancer Res*.

[37] Tang X, Mu J, Ma L, Tan Q, Wang J, Tan J, Zhang S (2021). IGFBP7 overexpression promotes acquired resistance to AZD9291 in non-small cell lung cancer. *Biochem Biophys Res Commun*.

[38] Pan Y, Lin S, Xing R, Zhu M, Lin B, Cui J, Li W, Gao J, Shen L, Zhao Y (2016). Epigenetic Upregulation of Metallothionein 2A by Diallyl Trisulfide Enhances Chemosensitivity of Human Gastric Cancer Cells to Docetaxel Through Attenuating NF-κB Activation. *Antioxidants & Redox Signaling*.

[39] Lin S, Lin B, Wang X, Pan Y, Xu Q, He JS, Gong W, Xing R, He Y, Guo L (2017). Silencing of ATP4B of ATPase H+/K+ Transporting Beta Subunit by Intragenic Epigenetic Alteration in Human Gastric Cancer Cells. *Oncology Research*.

[40] Bostick M, Kim JK, Estève PO, Clark A, Pradhan S, Jacobsen SE (2007). UHRF1 plays a role in maintaining DNA methylation in mammalian cells. *Science*.

[41] Liu X, Gao Q, Li P, Zhao Q, Zhang J, Li J, Koseki H, Wong J (2013). UHRF1 targets DNMT1 for DNA methylation through cooperative binding of hemi-methylated DNA and methylated H3K9. *Nat Commun*.

[42] Liu Q, Yu S, Zhao W, Qin S, Chu Q, Wu K (2018). EGFR-TKIs resistance via EGFR-independent signaling pathways. *Mol Cancer*.

[43] Lim SM, Syn NL, Cho BC, Soo RA (2018). Acquired resistance to EGFR targeted therapy in non-small cell lung cancer: Mechanisms and therapeutic strategies. *Cancer Treat Rev*.

[44] Nanjo S, Arai S, Wang W, Takeuchi S, Yamada T, Hata A, Katakami N, Okada Y, Yano S (2017). MET Copy Number Gain Is Associated with Gefitinib Resistance in Leptomeningeal Carcinomatosis of EGFR-mutant Lung Cancer. *Mol Cancer Ther*.

[45] Cappuzzo F, Jänne PA, Skokan M, Finocchiaro G, Rossi E, Ligorio C, Zucali PA, Terracciano L, Toschi L, Roncalli M (2009). MET increased gene copy number and primary resistance to gefitinib therapy in non-small-cell lung cancer patients. *Ann Oncol*.

[46] Nguyen HN, Cao NT, Van Nguyen TC, Le KND, Nguyen DT, Nguyen QT, Nguyen TT, Van Nguyen C, Le HT, Nguyen MT (2021). Liquid biopsy uncovers distinct patterns of DNA methylation and copy number changes in NSCLC patients with different EGFR-TKI resistant mutations. *Sci Rep*.

[47] Su SF, Liu CH, Cheng CL, Ho CC, Yang TY, Chen KC, Hsu KH, Tseng JS, Chen HW, Chang GC (2021). Genome-Wide Epigenetic Landscape of Lung Adenocarcinoma Links HOXB9 DNA Methylation to Intrinsic EGFR-TKI Resistance and Heterogeneous Responses. *JCO Precis Oncol*.

[48] Hou T, Ma J, Hu C, Zou F, Jiang S, Wang Y, Han C, Zhang Y (2019). Decitabine reverses gefitinib resistance in PC9 lung adenocarcinoma cells by demethylation of RASSF1A and GADD45β promoter. *Int J Clin Exp Pathol*.

[49] Zhao M, Zhang Y, Li J, Li X, Cheng N, Wang Q, Cai W, Zhao C, He Y, Chang J, Zhou C (2018). Histone deacetylation, as opposed to promoter methylation, results in epigenetic BIM silencing and resistance to EGFR TKI in NSCLC. *Oncol Lett*.

[50] Maunakea AK, Nagarajan RP, Bilenky M, Ballinger TJ, D'Souza C, Fouse SD, Johnson BE, Hong C, Nielsen C, Zhao Y (2010). Conserved role of intragenic DNA methylation in regulating alternative promoters. *Nature*.

[51] Dhanisha SS, Guruvayoorappan C, Drishya S, Abeesh P (2018). Mucins: Structural diversity, biosynthesis, its role in pathogenesis and as possible therapeutic targets. *Crit Rev Oncol Hematol*.

[52] Jonckheere N, Van Seuningen I (2018). Integrative analysis of the cancer genome atlas and cancer cell lines encyclopedia large-scale genomic databases: MUC4/MUC16/MUC20 signature is associated with poor survival in human carcinomas. *J Transl Med*.

[53] Moniaux N, Junker WM, Singh AP, Jones AM, Batra SK (2006). Characterization of Human Mucin MUC17. *Journal of Biological Chemistry*.

[54] Kitamoto S, Yamada N, Yokoyama S, Houjou I, Higashi M, Goto M, Batra SK, Yonezawa S (2011). DNA methylation and histone H3-K9 modifications contribute to MUC17 expression. *Glycobiology*.

[55] Yu WH, Wu E, Li Y, Hou HH, Yu SC, Huang PT, Kuo WH, Qi D, Yu CJ (2020). Matrix Metalloprotease-7 Mediates Nucleolar Assembly and Intra-nucleolar Cleaving p53 in Gefitinib-Resistant Cancer Stem Cells. *iScience*.

[56] Chen CH, Wang SW, Chen CW, Huang MR, Hung JS, Huang HC, Lin HH, Chen RJ, Shyu MK, Huang MC (2013). MUC20 overexpression predicts poor prognosis and enhances EGF-induced malignant phenotypes via activation of the EGFR-STAT3 pathway in endometrial cancer. *Gynecol Oncol*.

[57] Zhang H, Gao Q, Tan S, You J, Lyu C, Zhang Y, Han M, Chen Z, Li J, Wang H (2019). SET8 prevents excessive DNA methylation by methylation-mediated degradation of UHRF1 and DNMT1. *Nucleic Acids Res*.

[58] Kong X, Chen J, Xie W, Brown SM, Cai Y, Wu K, Fan D, Nie Y, Yegnasubramanian S, Tiedemann RL (2019). Defining UHRF1 Domains that Support Maintenance of Human Colon Cancer DNA Methylation and Oncogenic Properties. *Cancer Cell*.

[59] Ahmad A, Ginnebaugh KR, Li Y, Padhye SB, Sarkar FH (2015). Molecular targets of naturopathy in cancer research: bridge to modern medicine. *Nutrients*.

[60] Scheiermann E, Puppa MA, Rink L, Wessels I (2022). Zinc Status Impacts the Epidermal Growth Factor Receptor and Downstream Protein Expression in A549 Cells. *Int J Mol Sci*.

[61] Barth LM, Rink L, Wessels I (2020). Increase of the Intracellular Zinc Concentration Leads to an Activation and Internalisation of the Epidermal Growth Factor Receptor in A549 Cells. *Int J Mol Sci*.

[62] Mincione G, Di Marcantonio MC, Tarantelli C, Savino L, Ponti D, Marchisio M, Lanuti P, Sancilio S, Calogero A, Di Pietro R, Muraro R (2016). Identification of the zinc finger 216 (ZNF216) in human carcinoma cells: a potential regulator of EGFR activity. *Oncotarget*.

