# Supplementary material for: Acquired resistance to EGFR-TKIs in NSCLC mediates epigenetic downregulation of MUC17 by facilitating NF-κB activity via UHRF1/DNMT1 complex
Source: Int J Biol Sci. 2023 Jan 9;19(3):832–51. doi: 10.7150/ijbs.75963 (PMC9910003; doi:10.7150/ijbs.75963)
Supplement: Supplementary file 1 — Supplementary figures and tables. [file ijbsv19p0832s1.pdf]

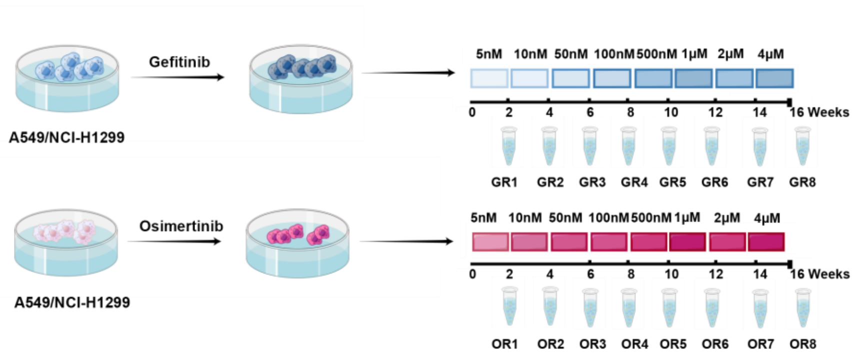

Supplementary Figure 1. A549 and NCI-H1299 cells were treated with gefitinib/osimertinib at the indicated concentrations for 16 weeks to establish gefitinib-resistant cells (GR1-8) and osimertinib-resistant cells (OR1-8).

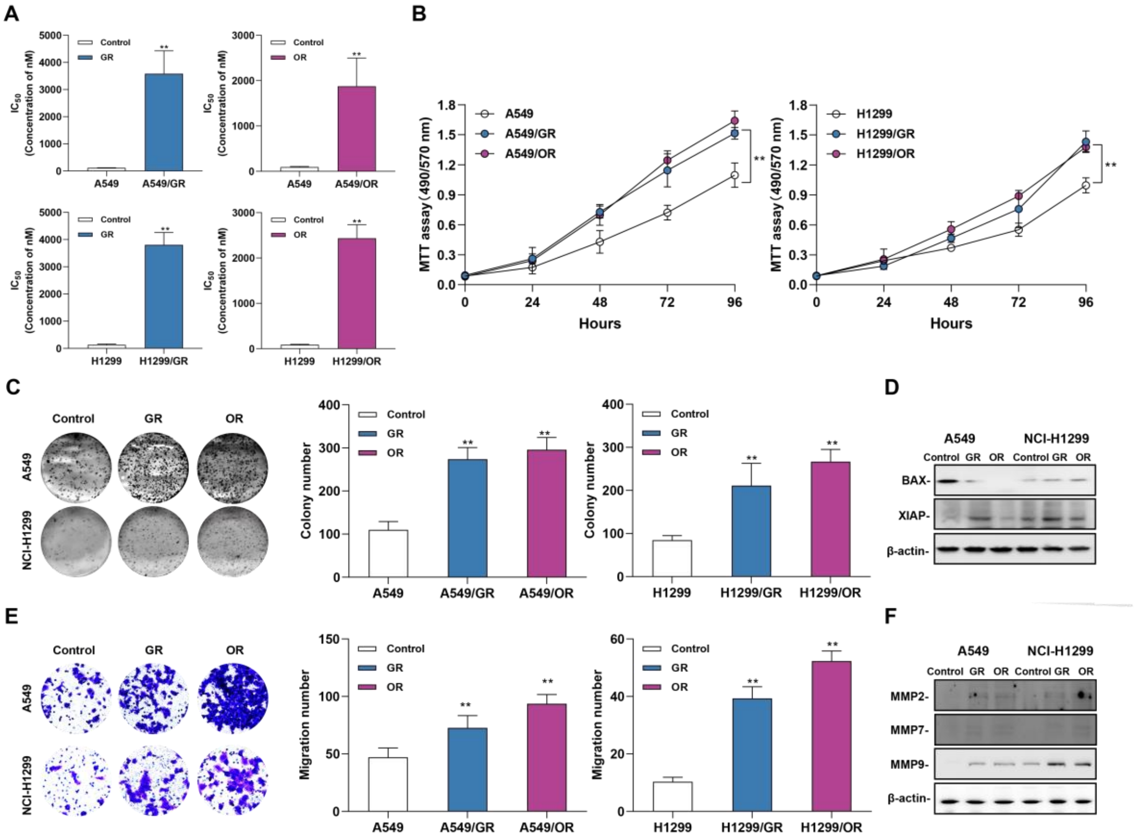

Supplementary Figure 2. The Biological function of gefitinib/osimertinib-resistant A549 and NCI-H1299 cells. (A) IC50 analysis of gefitinib /Osimertinib-resistant cells. **(B)** MTT assay performed to monitor the viability of cells over 4 days. **(C)** Quantification of the total number of colonies formed for 2 weeks. **(D)** Western blotting analysis of the protein levels of BAX and XIAP in GR and OR. β-actin serves as the loading control. **(E)** Quantification of cell motility by Transwell assay. **(F)** Western blotting analysis of the protein levels of MMP2, MMP7 and MMP9 in GR and OR cells. β-actin serves as the loading control. Data are presented as the mean ± standard deviation (n=3). One-way ANOVA with Tukey's post-hoc test was performed. *$p < 0.05$, ** $p < 0.01$, significant differences versus control.

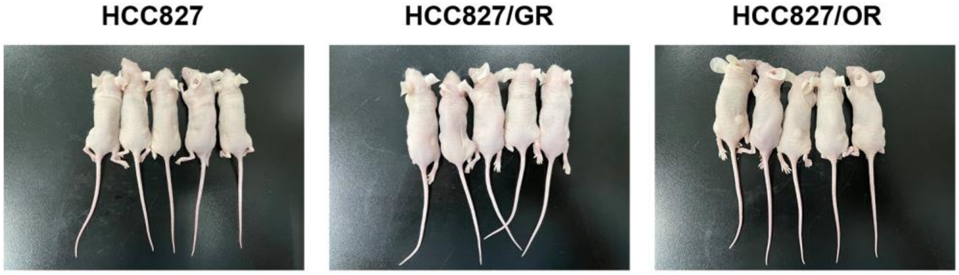

Supplementary Figure 3. The effect of HCC827, HCC827/GR and HCC827/OR on tumorigenicity *in vivo.* Xenograft tumor-bearing mice were sacrificed on day 30 and photographed.

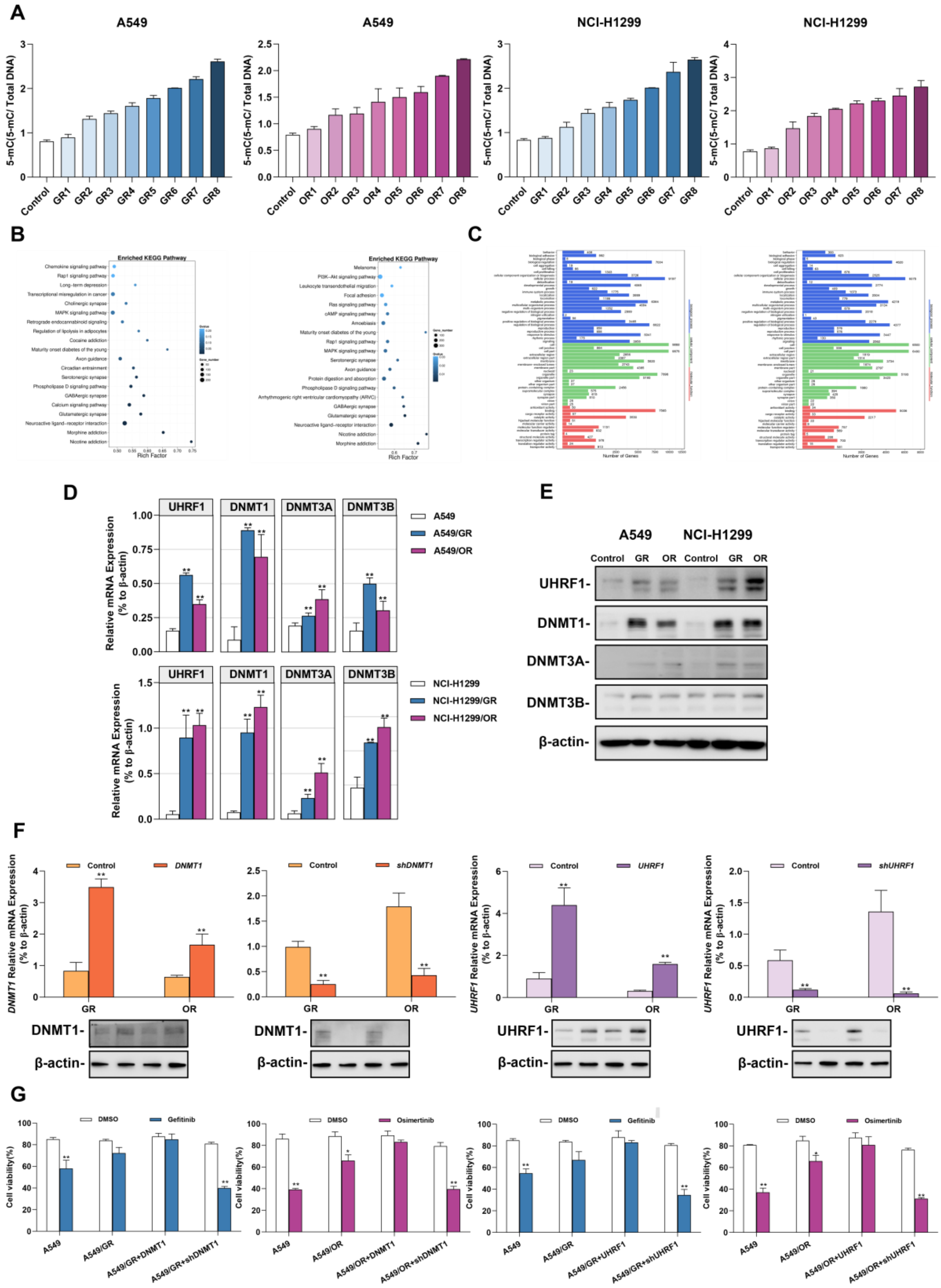

Supplementary Figure 4. Alterations in DNA methylation in gefitinib/osimertinib-resistant A549 and NCI-H1299 cells. (A) ELISA analysis of global DNA methylation in

gefitinib (GR1-8)/osimertinib-resistant cells (OR1-8). **(B)** KEGG pathway enrichment for the genes overlapping with DMRs in the promoter region of A549 *vs* A549/GR (A) or A549 *vs* A549/OR (B). **(C)** Gene ontology enrichment analysis of the differentially methylated sites (DMSs) in the promoter region of A549 *vs* A549/GR or A549 *vs* A549/OR, based on the biological process (blue), cellular components (green) and molecular function (red) categories of the annotated genes. **(D)** and **(E)** RT–qPCR and Western blots analysis of the expression of DNA methyltransferases (*DNMT1*, *DNMT3A* and *DNMT3B*) and *UHRF1* in GR and OR. (D) RT–qPCR, (E) Western blots. **(F)** *DNMT1* or *UHRF1* was overexpressed or knocked down in A549/GR and A549/OR. RT–qPCR and Western blotting analysis of DNMT1 and UHRF1. β-actin as sample loading control. **(G)** Cell viability was measured by MTT in gefitinib/osimertinib-resistant cells treatment with gefitinib (2μM) / osimertinib (1μM). Data are presented as the mean ± standard deviation (n=3). *$p < 0.05$, ** $p < 0.01$, significant differences versus control (D, one-way ANOVA with Tukey's post hoc test; F and G, two-tailed Student's t-test).

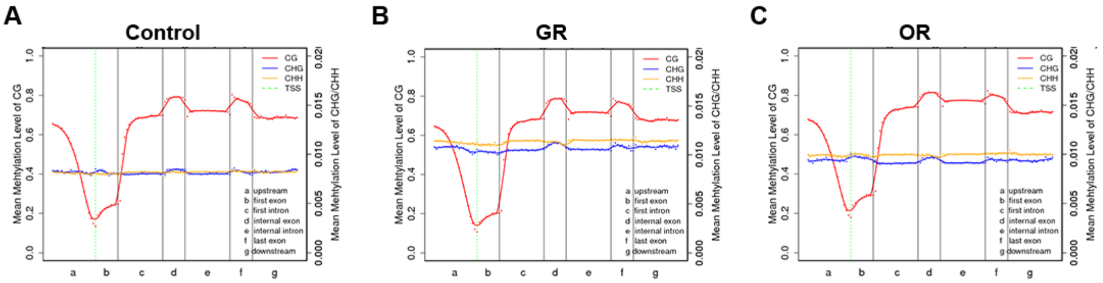

Supplementary Figure 5. DNA Methylation Patterns Across the Entire Transcriptional Units at Whole Genome Level. The canonical gene structure is defined by 7 different features, denoted by the x-axis. The length of each feature was normalized and divided into equal numbers of bins. Each dot denotes the mean methylation level per bin and the respective lines denote the 5-bin moving average. Each feature was analyzed separately for the numbers listed in the table below the figure. The green vertical line indicates the mean location of the transcription start sites. **(A)** Control. **(B)** A549/GR. **(C)** A549/OR.

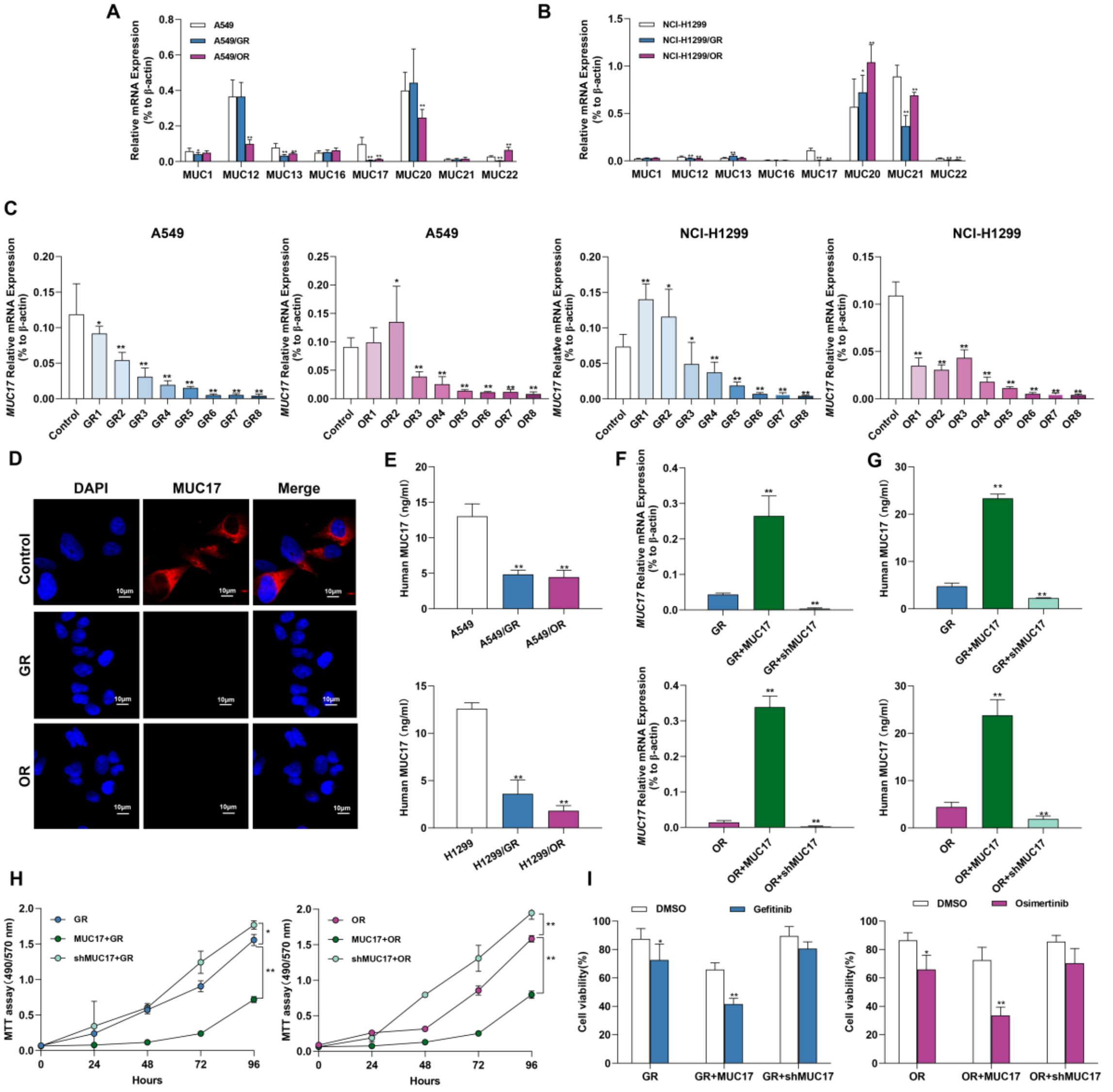

Supplementary Figure 6. Gefitinib and osimertinib resistance inhibited MUC17 expression, enhancing lung cancer cell proliferation in A549 and NCI-H1299 cells. **(A)** and **(B)** RT–qPCR showing the mRNA expression of membrane-bound mucins (*MUCs*) in gefitinib/osimertinib-resistant A549(A) and NCI-H1299(B) cells. Each gene was compared with its own untreated control after normalization to β-actin. **(C)** RT–qPCR of *MUC17* expression in GR1-8 and OR1-8 cells. **(D)** Immunofluorescence staining of MUC17 (red) in A549, A549/GR and A549/OR cells. DAPI-stained nuclei were shown as blue. Scale bar: *10 μM.* **(E)** ELISA analysis of MUC17 expression in gefitinib/osimertinib-resistant A549 and NCI-H1299 cells. **(F)** and **(G)** A549/GR and A549/OR cells stably expressing ectopic truncated *MUC17* or *shMUC17*. **(F)** The mRNA

expression level of *MUC17* was assessed using RT–qPCR. **(G)** The protein expression level of MUC17 was assessed using ELISA. **(h)** MTT showing cell viability. **(I)** MTT showing cell viability treatment with gefitinib (2μM) /osimertinib (1μM). Data are presented as the mean ± standard deviation (n=3). One-way ANOVA with Tukey's post-hoc test was performed for A, B, C, E, F, G and H, two-tailed Student's t test was used for I. **p* < 0.05, ***p* < 0.01, significant differences from the control.

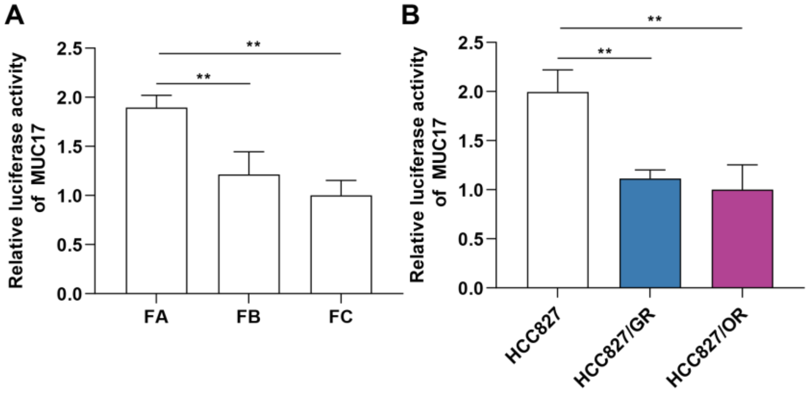

Supplementary Figure 7. The promoter activity of MUC17 in response to TKI resistance. The high frequency binding sites of transcription factors in the *MUC17* promoter region are divided into three segments (FA-FC). These sequences included progressive deletions from upstream of the transcriptional start site, termed FA (− 846 bp), FB (− 646 bp), and FC (− 446 bp). (A) Three *MUC17* promoter fragments were transferred into HCC827 cells and detected using luciferase assays. (B) Luciferase reporter assays showing the activity of *MUC17* promoter region (FA) in HCC827, HCC827/GR and HCC827/OR.

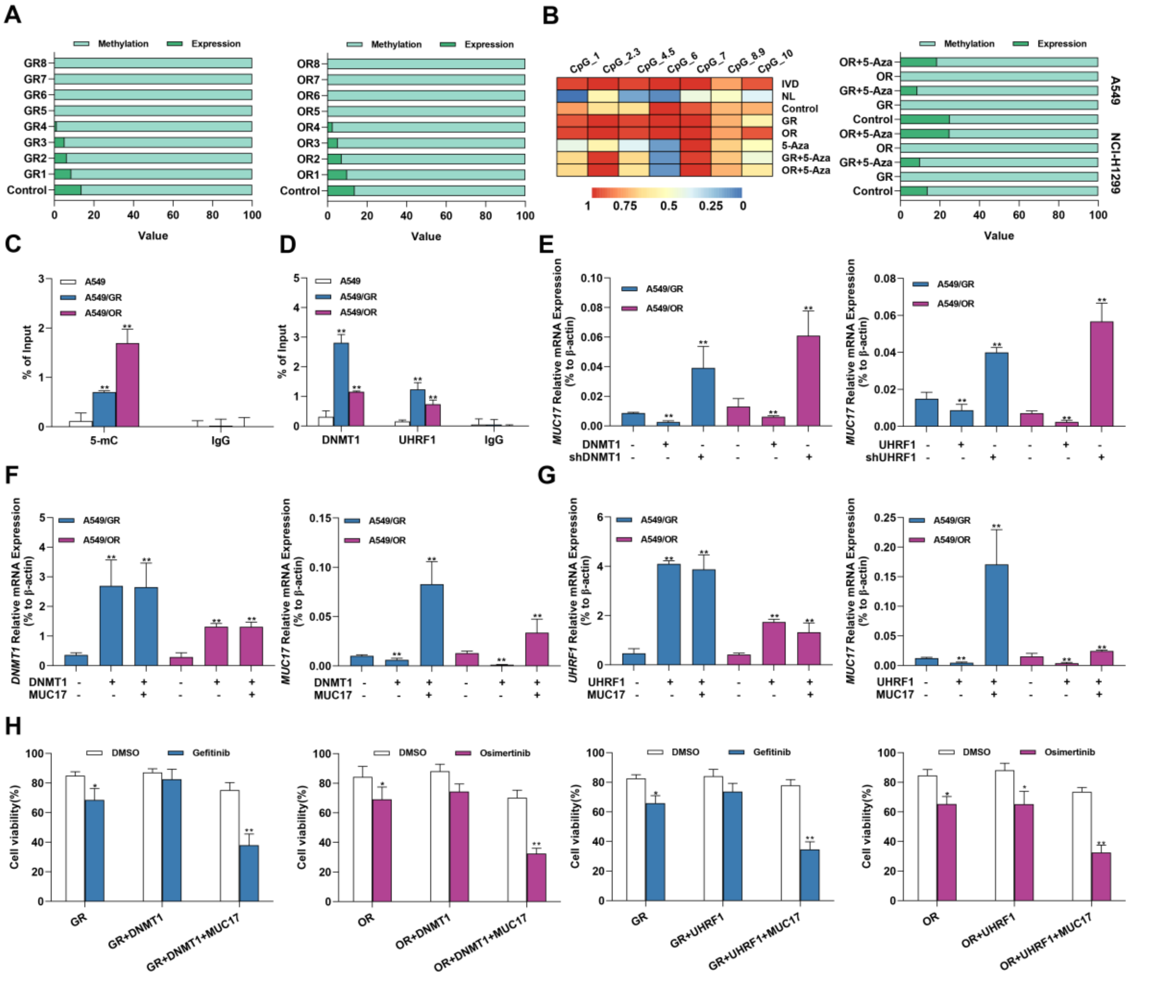

Supplementary Figure 8. Gefitinib/osimertinib resistance-mediated epigenetic downregulation of *MUC17* through promoter hypermethylation in A549 and NCI-H1299 cells. (A) 100% stacked bar graph showing the time course association of *MUC17* expression with its promoter methylation in GR1-8 and OR1-8, RT–qPCR and MS-qPCR were used to detect *MUC17* expression and methylation with the primers listed in **Supplementary Table 1.** The comparison between methylation and expression was visualized by a 100% stacked bar graph. **(B)** MassARRAY and MSP analysis of *MUC17* methylation in A549, A549/GR and A549/OR or treatment with 5-aza-2'-deoxycytidine (5-Aza, 5 μM, 96 h). IVD: in vitro methylated DNA served as a positive control; NL: normal blood lymphocyte DNA served as a negative control. **(C)** MeDIP-qPCR analysis of anti-5-mC enriched DNA fragments at the *MUC17* promoter region in A549, A549/GR and A549/OR cells. The specific enrichment is represented as the percentage of input (% of input) after normalization to the nonspecific IgG control. **(D)** ChIP-qPCR was used to

analyze the immunoprecipitated DNA fraction with primers covering the *MUC17* promoter region. The normalized enrichment was represented as "% of input". **(E)** RT–qPCR analysis of *MUC17* in A549/GR and A549/OR stably expressing ectopic *DNMT1* or *shDNMT1*, *UHRF1* or *shUHRF1*. **(F)** RT–qPCR analysis of *DNMT1* and *MUC17* in A549/GR and A549/OR stably expressing ectopic *DNMT1* and/or *MUC17*. **(G)** RT–qPCR analysis of *UHRF1* and *MUC17* in A549/GR and A549/OR stably expressing ectopic *UHRF1* and/or *MUC17*. **(H)** MTT showing cell viability treatment with gefitinib (2μM) /osimertinib (1μM). Data are presented as the mean ± standard deviation (n=3). One-way ANOVA with Tukey's post-hoc test was performed. **p* < 0.05, ** *p* < 0.01, significant differences versus control.

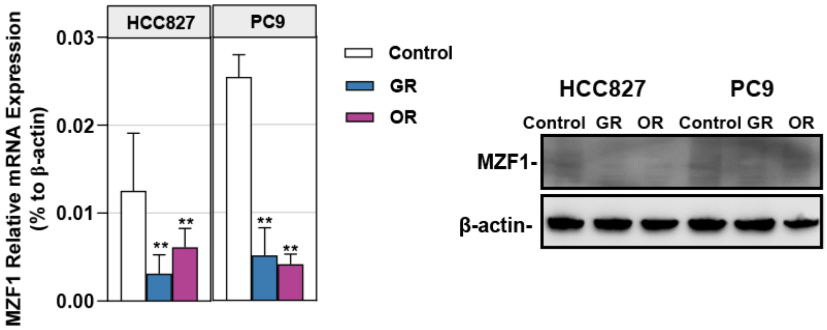

Supplementary Figure 9. RT–qPCR and Western blots analysis of the expression of *MZF1* in GR and OR.

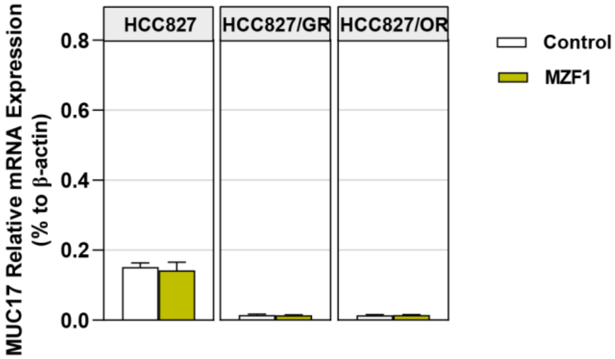

Supplementary Figure 10. RT–qPCR analysis of the expression of *MUC17* in HCC827, HCC827/GR and HCC827/OR stably expressing ectopic *MZF1*.

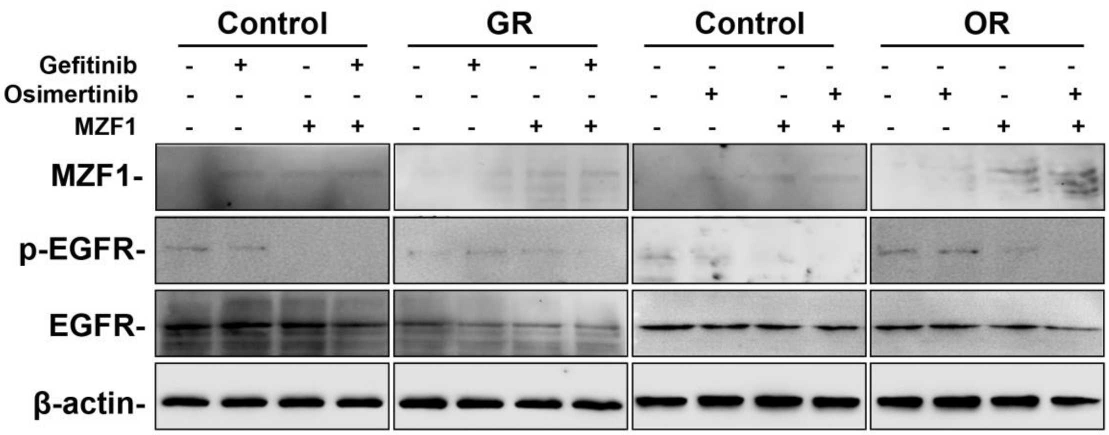

Supplementary Figure 11. Western blotting analysis of MZF1, p-EGFR, EGFR in HCC827, HCC827/GR and HCC827/OR were treatment with Gefitinib, Osimertinib and/or transfect with MZF1.

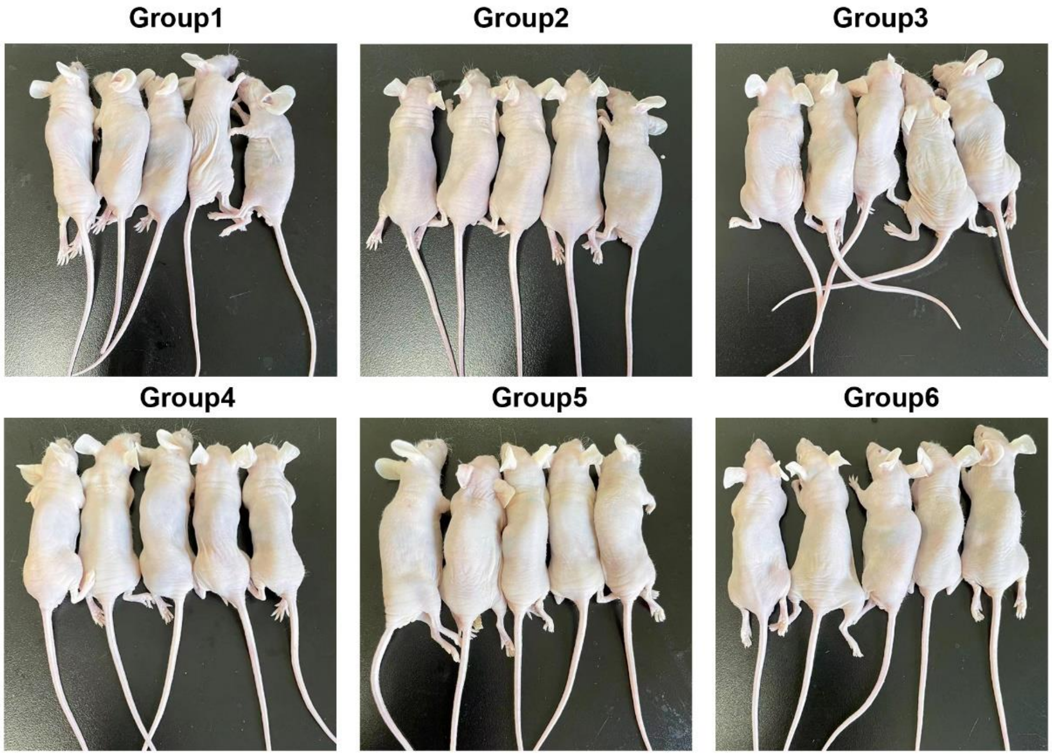

Supplementary Figure 12. The effect of A549, A549/GR and A549/OR on tumorigenicity *in vivo.* Xenograft tumor-bearing mice were sacrificed on day 24 and photographed.

Supplementary table 1. Oligo nucleotides

NCBI Accession Number	Target gene (size)	Primer ID	Sequence (5' to 3')	Oligo use for
NM_001040105.1	MUC17 (91bp)	F	GGGCCAGCATAGCTTCGA	RT-qPCR
		R	GCTACAGGAATTGTGGGAGTTGA	
NM_001101	β-actin (130bp)	F	TTAGTTGCGTTACACCCTTTC	
		R	ACCTTCACCGTTCCAGTTT	
NM_001018016.2	MUC1 (116bp)	F	TACAGCTACCACAGCCCCTA	
		R	AGCTGGGCACTGAACTTCTC	
NM_001164462.1	MUC12 (266bp)	F	CTCGTGTATGGGATCGTGGG	
		R	AGCTCTGTGCCAGAGTCAAC	
NM_033049.3	MUC13 (107bp)	F	AAGAATGTGGAACCCGCCAT	
		R	CCCGGAGGCCAGATCTTTAC	
NM_024690.2	MUC16 (97bp)	F	CACCTGGGATGTCCACCTTG	
		R	CGACGGTTATAACTGCTGGTGGT	
NM_152673.3	MUC20 (106bp)	F	CAAGATCACAACCTCAGCGA	
		R	ACCTCCATTTTCACCTGCAC	
NM_001010909.5	MUC21 (124bp)	F	TAGCACCTCTGCCAACACTG	
		R	GGTCACGCTGGACCCT	
NM_001198815.1	MUC22 (239bp)	F	TGGCCTCTACTTCGGCCTTA	
		R	GGTGGAGGCCACGATAGTTT	
NM_001379.4	DNMT1 (104 bp)	F	CCAAAGAACCAACACCCAAAC	
		R	CTCATCTTTCTCGTCTCCATCTTC	
NM_001320892.2	DNMT3A (95 bp)	F	ACGATTGCTAGACTGGGATAATG	
		R	AGTAAGCAGGCCAGGTAGA	
NM_175850.3	DNMT3B (98 bp)	F	GGAGCCACGACGTAACAAATA	
		R	GTAAACTCTAGGCATCCGTCATC	
NM_001290050.2	UHRF1 (108 bp)	F	AAATGGCCTCAAGGGGACTC	
		R	CACTTGCACGTGACTTCGTG	
NM_198055.2	MZF1 (162 bp)	F	ATGCAGGAATCACCACTGGG	
		R	AAAGATCTGGTCCAGCACGG	
NM_001045868.1	IκB-α (197bp)	F	CGTCTTATTGTGGTAGGATCAGC	
		R	ACCACTGGGGTCAGTCACTC	
NC_000007.14 (MUC17 Promoter region)	MUC17-U (273bp)	F	GTTGATAATTTTTATGTTTATGGGTTGTT	MSP
		R	CTAACCTTAACATCAAAACTCTAAACACA	
	MUC17-M (269bp)	F	GTTGATAATTTTTATGTTTATGGGTTGTC	
		R	CCTTAACATCGAAACTCTAAACACG	
	MUC17 (439bp)	F	GGAAAGGAGGAGGGTATTATTTGTA	MassARRAY
		R	CCCAAACATAACCATATCTTCAAAA	
	MUC17 (269bp)	F	TCTGTTGGGGTGATAAAGCTGA	MeDIP/ChIP-qPCR
		R	TCTGTTGGGGTGATAAAGCTGA	
	MUC17-FA	F	TCCCCCGGGTtattttatttatttatt	

		R	CCGCTCGAGCCTGTTCTGCAGCAGCTT	
	MUC17-FB	F	TCCCCCGGGTtcatcatgttggccagg	Luciferase assay
		R	CCGCTCGAGCCTGTTCTGCAGCAGCTT	
	MUC17-FC	F	TCCCCCGGGCagtccctcttgcccatc	
		R	CCGCTCGAGCCTGTTCTGCAGCAGCTT	
NC_037348.1 (IκB-α Promoter region)	IκB-α (283bp)	F	AATCCCTACGCCCAGCCATC	ChIP-qPCR
		R	CTTTTTGATGAGCGCCGAGTG	
	IκB-α	F	GGAAGATCTGAAAGCAAATCCCTA	Luciferase assay
		R	CCCAAGCTTCGGACGAGCTGCGGG	
	Positive control	F	GGCGTAATGGAATGCTTGA	MeDIP-qPCR
		R	CCTCGCCTAGTCTGGAAGC	
	Negative control	F	GGTTCAGTTTATTGTCCTAAAATCAG	
		R	TCAGCCAGACCAAAGCAAAT	

Note: F: forward, R: reverse, M: methylated, U: unmethylated

Supplementary table 2. Antibodies used in this study.

Antibody	Type	Vendor	Cat#	WB	IF	IHC	ChIP or MeDIP
Anti-MUC17	rabbit polyclonal	Sigma Aldrich	HPA031634	1:100	1:25	1:25	NA
Anti-Bax	rabbit polyclonal	Proteintech	50599-2-Ig	1:800	NA	NA	NA
Anti-XIAP	rabbit polyclonal	Proteintech	10037-1-Ig	1:800	NA	NA	NA
Anti-MMP2	rabbit polyclonal	Proteintech	10373-2-AP	1:800	NA	NA	NA
Anti-MMP7	rabbit polyclonal	Proteintech	10374-2-AP	1:800	NA	NA	NA
Anti- MMP9	rabbit polyclonal	Proteintech	10375-2-AP	1:800	NA	NA	NA
Anti-EGFR	rabbit polyclonal	Proteintech	66455-1-Ig	1:3000	NA	NA	NA
Anti-NF-κB p65	rabbit monoclonal	Abcam	ab32536	1:1000	1:200	NA	NA
Anti-phospho-NF-κB p65	rabbit monoclonal	Abcam	ab76302	1:1000	NA	NA	NA
Anti-IκB-α	rabbit monoclonal	Abcam	ab32518	1:1000	NA	NA	NA
Anti-phospho-IκB-α	rabbit monoclonal	Abcam	ab133462	1:1000	NA	NA	NA
Anti-Lamin A/C	rabbit polyclonal	Proteintech	10298-1-AP	1:6000	NA	NA	NA
Anti-β-actin	mouse monoclonal	Sigma Aldrich	A4551	1:10000	NA	NA	NA
Anti-ATP1A1	mouse monoclonal	Proteintech	55187-1-AP	1:5000	NA	NA	NA
Anti-DNMT3A	mouse monoclonal	Active Motif	39206	1:1000	NA	NA	NA
Anti-DNMT3B	mouse monoclonal	Abcam	ab79822	1:3000	NA	NA	NA
Anti-DNMT1	mouse monoclonal	Abcam	ab188453	1:1000	1:200	1:1000	2 µg
Anti-UHRF1	rabbit monoclonal	Active Motif	61341	1:2000	1:200	NA	2 µg
Anti-MZF1	rabbit monoclonal	Abcam	ab64866	1:800	1:100	1:100	2 µg
Anti-5-methylcytidine (5mC)	rabbit monoclonal	Active Motif	91311	NA	1:2000	NA	0.5 µg
CL488 – conjugated Affinipure Goat Anti-Mouse IgG(H＋L)		Proteintech	SA00013-1	NA	1:200	NA	NA
CL594 – conjugated Goat Anti-Rabbit IgG(H+L)		Proteintech	SA00013-4	NA	1:200	NA	NA
Goat Anti-Mouse IgG H+L (HRP)		Abcam	ab6789	1:2500	NA	NA	NA
Goat Anti-Rabbit IgG H+L (HRP)		Abcam	ab6721	1:2500	NA	NA	NA

Supplementary table 3. Reagents used in this study.

Reagents	Vendor	Cat#	Concentration
Gefitinib	Sigma Aldrich	SML1657	5nM-4 μM
Osimertinib	MCE	HY-15572	1nM-4μM
BAY11-7082	MCE	HY-13453	8 μM
5-aza-2'-deoxycytidine	Sigma Aldrich	189825	5 μM

Supplementary table 4. Summary of patients' clinical information (BALF, n=57).

Sample ID	Sex	Age	Pathological type	T	N	M	Clinical stage	EGFR mutation
C1	Male	70	chronic pneumonia					
C2	Female	63	chronic pneumonia					
C3	Male	38	chronic pneumonia					
C4	Female	72	chronic pneumonia					
C5	Male	86	chronic pneumonia					
C6	Male	69	chronic pneumonia					
C7	Female	50	chronic pneumonia					
C8	Male	75	chronic pneumonia					
C9	Female	55	chronic pneumonia					
C10	Female	54	chronic pneumonia					
B1	Female	65	Lung adenocarcinoma	1	0	0	I	19DEL
B2	Male	66	Lung adenocarcinoma	4	2	0	III	L858R
B3	Female	75	Lung adenocarcinoma	1	0	1	IV	19DEL
B4	Male	75	Lung adenocarcinoma	1	0	0	I	19DEL
B5	Male	68	Lung adenocarcinoma	1	0	0	I	L858R
B6	Male	62	Lung squamous cell carcinomas	2	2	0	III	19DEL
B7	Female	63	Lung adenocarcinoma	1	0	0	I	L858R
B8	Male	62	Lung adenocarcinoma	1	0	0	I	19DEL
B9	Male	63	Lung adenocarcinoma	1	0	0	I	L858R
B10	Male	57	Lung adenocarcinoma	1	0	0	I	19DEL
B11	Female	68	Lung adenocarcinoma	1	0	0	I	L858R
B12	Female	65	Lung adenocarcinoma	1	0	0	I	19DEL
B13	Female	62	Lung adenocarcinoma	1	0	0	I	19DEL
B14	Male	63	Lung adenocarcinoma	2	2	0	I	19DEL
B15	Female	58	Lung adenocarcinoma	1	0	0	III	19DEL
B16	Male	57	Lung adenocarcinoma	1	0	0	I	L858R
B17	Male	40	Lung adenocarcinoma	1	0	0	I	19DEL
B18	Male	65	Lung adenocarcinoma	1	0	0	I	19DEL
B19	Male	76	Lung squamous cell carcinomas	1	0	0	I	19DEL
B20	Male	73	Lung squamous cell carcinomas	2	0	0	I	19DEL
B21	Female	31	Lung adenocarcinoma	1	0	0	I	19DEL
B22	Female	55	Lung adenocarcinoma	2	0	1	IV	T790M
G1	Female	70	Lung adenocarcinoma	3	3	0	IV	19DEL
G2	Male	66	Lung adenocarcinoma	2	2	0	III	19DEL
G3	Female	45	Lung adenocarcinoma	3	3	1	IV	19DEL
G4	Female	76	Lung adenocarcinoma	3	2	0	III	L858R
G5	Female	37	Lung adenocarcinoma	3	2	1	IV	19DEL
G6	Female	57	Lung adenocarcinoma	2	3	1	IV	19DEL
G7	Female	69	Lung adenocarcinoma	4	3	1	IV	L858R
G8	Male	63	Lung squamous cell carcinomas	2	1	0	II	19DEL
G9	Male	65	Lung adenocarcinoma	1	0	0	I	L858R
G10	Female	62	Lung adenocarcinoma	4	3	1	IV	19DEL
G11	Male	64	Lung adenocarcinoma	4	2	1	IV	L858R
G12	Female	58	Lung adenocarcinoma	2	2	0	III	19DEL
G13	Male	54	Lung adenocarcinoma	3	0	1	IV	19DEL
O1	Male	64	Lung adenocarcinoma	4	3	1	IV	T790M
O2	Female	63	Lung adenocarcinoma	4	0	1	IV	19DEL
O3	Female	54	Lung adenocarcinoma	3	3	0	III	T790M
O4	Female	70	Lung adenocarcinoma	2	3	1	IV	T790M
O5	Female	61	Lung adenocarcinoma	2	2	1	IV	T790M
O6	Female	66	Lung adenocarcinoma	2	2	0	III	19DEL
O7	Female	62	Lung adenocarcinoma	3	3	0	IV	T790M
O8	Male	58	Lung adenocarcinoma	2	2	0	III	T790M
O9	Female	67	Lung adenocarcinoma	3	3	1	IV	T790M
O10	Male	51	Lung adenocarcinoma	2	1	1	IV	T790M
O11	Female	61	Lung adenocarcinoma	2	2	1	IV	19DEL
O12	Female	66	Lung adenocarcinoma	4	3	1	IV	T790M

Supplementary table 5. A summary of the whole genome bisulfite sequencing reads from the DNA extracted from A549 cells

	Clean Reads	Mapped Reads	Mapping Rate (%)	Uniquely Mapped Reads	Uniquely Mapping Rate (%)	Bisulfite Conversion Rate (%)
Control	1189716332	1117738630	93.95	1065339049	89.55	99.28
GR	1053043860	984463005	93.49	932839014	88.59	99.17
OR	1022414092	970366657	94.91	924850608	90.46	99.33

Supplementary table 6. KEGG pathway enrichment for the genes overlapping with DMRs from A549 vs A549/GR

Pathway	Gene number	Background gene number	Rich Factor	Q value
Nicotine addiction	47	63	0.746032	3.04E-05
Morphine addiction	79	121	0.652893	3.04E-05
Neuroactive ligand-receptor interaction	212	404	0.524752	1.29E-03
Glutamatergic synapse	85	147	0.578231	5.26E-03
Calcium signaling pathway	134	248	0.540323	5.26E-03
GABAergic synapse	70	119	0.588235	8.16E-03
Phospholipase D signaling pathway	102	187	0.545455	1.58E-02
Serotonergic synapse	75	133	0.56391	2.20E-02
Circadian entrainment	72	130	0.553846	4.88E-02
Axon guidance	128	251	0.50996	7.55E-02
Maturity onset diabetes of the young	22	33	0.666667	9.59E-02
Cocaine addiction	36	60	0.6	9.59E-02
Regulation of lipolysis in adipocytes	44	77	0.571429	1.24E-01
Retrograde endocannabinoid signaling	90	174	0.517241	1.25E-01
MAPK signaling pathway	181	373	0.485255	1.25E-01
Cholinergic synapse	72	136	0.529412	1.25E-01
Transcriptional misregulation in cancer	170	350	0.485714	1.35E-01
Long-term depression	42	75	0.56	1.72E-01
Rap1 signaling pathway	140	288	0.486111	2.13E-01
Chemokine signaling pathway	124	253	0.490119	2.13E-01

Supplementary table 7. KEGG pathway enrichment for the genes overlapping with DMRs from A549 vs A549/OR

Pathway	Gene number	Background gene number	Rich Factor	Q value
Morphine addiction	87	121	0.719008264	7.53E-06
Nicotine addiction	48	63	0.761904762	2.56E-04
Neuroactive ligand-receptor interaction	236	404	0.584158416	2.56E-04
Glutamatergic synapse	96	147	0.653061224	4.60E-04
GABAergic synapse	77	119	0.647058824	4.73E-03
Arrhythmogenic right ventricular cardiomyopathy (ARVC)	70	109	0.642201835	1.15E-02
Phospholipase D signaling pathway	112	187	0.598930481	1.20E-02
Protein digestion and absorption	263	480	0.547916667	1.37E-02
Axon guidance	145	251	0.577689243	1.37E-02
Serotonergic synapse	82	133	0.616541353	1.50E-02
MAPK signaling pathway	207	373	0.554959786	1.70E-02
Rap1 signaling pathway	163	288	0.565972222	1.76E-02
Maturity onset diabetes of the young	25	33	0.757575758	1.78E-02
Amoebiasis	245	450	0.544444444	1.93E-02
cAMP signaling pathway	155	274	0.565693431	1.93E-02
Ras signaling pathway	173	311	0.556270096	2.71E-02
Focal adhesion	258	480	0.5375	3.19E-02
Leukocyte transendothelial migration	94	160	0.5875	3.20E-02
PI3K-Akt signaling pathway	321	607	0.528830313	3.20E-02
Melanoma	53	84	0.630952381	3.20E-02
